# Volcanic glass from the 1.8 ka Taupō eruption (New Zealand) detected in Antarctic ice at ~ 230 CE

**DOI:** 10.1038/s41598-023-42602-3

**Published:** 2023-10-09

**Authors:** Stephen B. Piva, Simon J. Barker, Nels A. Iverson, V. Holly L. Winton, Nancy A. N. Bertler, Michael Sigl, Colin J. N. Wilson, Nelia W. Dunbar, Andrei V. Kurbatov, Lionel Carter, Bruce L. A. Charlier, Rewi M. Newnham

**Affiliations:** 1 School of Geography, Environment and Earth Sciences, Victoria University of Wellington, P.O. Box 600, Wellington, 6140 New Zealand; 2 New Mexico Bureau of Geology and Mineral Resources, New Mexico Institute of Mining and Technology, 801 Leroy Place, Socorro, NM 87801 USA; 3 Antarctic Research Centre, Victoria University of Wellington, P. O. Box 600, Wellington, 6140 New Zealand; 4 GNS Science, National Isotope Centre, PO Box 30-368, Lower Hutt, 5040 New Zealand; 5 Climate and Environmental Physics, Physics Institute and Oeschger Centre for Climate Change Research, University of Bern, Hochschulstrasse 4, 3012 Bern, Switzerland; 6 Climate Change Institute, School of Earth and Climate Sciences, University of Maine, 168 College Avenue, Orono, ME 04469 USA

**Keywords:** Natural hazards, Cryospheric science

## Abstract

Chemical anomalies in polar ice core records are frequently linked to volcanism; however, without the presence of (crypto)tephra particles, links to specific eruptions remain speculative. Correlating tephras yields estimates of eruption timing and potential source volcano, offers refinement of ice core chronologies, and provides insights into volcanic impacts. Here, we report on sparse rhyolitic glass shards detected in the Roosevelt Island Climate Evolution (RICE) ice core (West Antarctica), attributed to the 1.8 ka Taupō eruption (New Zealand)—one of the largest and most energetic Holocene eruptions globally. Six shards of a distinctive geochemical composition, identical within analytical uncertainties to proximal Taupō glass, are accompanied by a single shard indistinguishable from glass of the ~25.5 ka Ōruanui supereruption, also from Taupō volcano. This double fingerprint uniquely identifies the source volcano and helps link the shards to the climactic phase of the Taupō eruption. The englacial Taupō-derived glass shards coincide with a particle spike and conductivity anomaly at 278.84 m core depth, along with trachytic glass from a local Antarctic eruption of Mt. Melbourne. The assessed age of the sampled ice is 230 ± 19 CE (95% confidence), confirming that the published radiocarbon wiggle-match date of 232 ± 10 CE (2 SD) for the Taupō eruption is robust.

## Introduction

Ice cores recovered from Antarctica contain exceptional continuous climate records extending back to ~800 ka^1^, with accompanying highly resolved geochemical and particulate records that chronicle past volcanism^2–5^. Volcanic signals related to aerosol deposition and physical traces in ice cores provide the opportunity to date, constrain, and link palaeoarchives, enabling the assessment of environmental and climatic impacts across regional and global scales^6–9^. The volcanically-produced sulfur (sulfate) measured in ice cores (i.e., non-sea-salt sulfur [nssS]) is used as a record of regional and global volcanism^2,5,6,9,10^, and provides insight into any potential volcanogenic climate forcing, as well as eruption plume extent and dynamics^7,11^. However, volcanic sulfur anomalies, irrespective of their apparently close temporal coincidence with documented eruptions, cannot unambiguously pinpoint the source eruptions, particularly in the prehistoric record. Unsupported linkages may have consequences for the precision and accuracy of ice core chronologies and evaluation of volcanic impacts^8^.

To overcome this problem, the geochemical characterisation and fingerprinting of visible (tephra) and microscopic (cryptotephra) layers of volcanic ash is evolving as an invaluable tool for correlating palaeoarchives (including glacial ice, and terrestrial and marine sediments), that can enable volcanic glass deposits to be attributed to a specific time-controlled eruptive source^12^. In ice cores from polar areas in both hemispheres, correlated tephras and (more recently) cryptotephras are used as valid high-precision climate-independent chronological markers, where annual layers can and cannot be resolved^8,13–16^. Englacial tephra records from Antarctic ice cores are dominated by regional volcanic sources, which have produced numerous explosive eruptions, typically of alkalic compositions, over the past 500,000 years^17^. This dominance in part reflects the challenge of finding and identifying tephra particles derived from non-Antarctic sources, and in part the large uncertainties in the age of prehistoric eruptions and distribution of their products^3,13,18–20^. While some tephra horizons derived from non-Antarctic eruptions are present and recognisable, small particle sizes (<10 µm) usually limit quantitative geochemical analysis and subsequent correlation to an eruptive source^21^. Nevertheless, recent progress in analytical capabilities and innovative preparation procedures has supported the isolation and geochemical fingerprinting of eruptive material associated with volcanic signals in polar ice cores^13,14,22,23^. These developments allow further direct correlations between different intercontinental sedimentary records to be made and provide valuable independent time-stratigraphic markers for ice core chronologies^8,10,13,23–25^.

**Figure 1:**
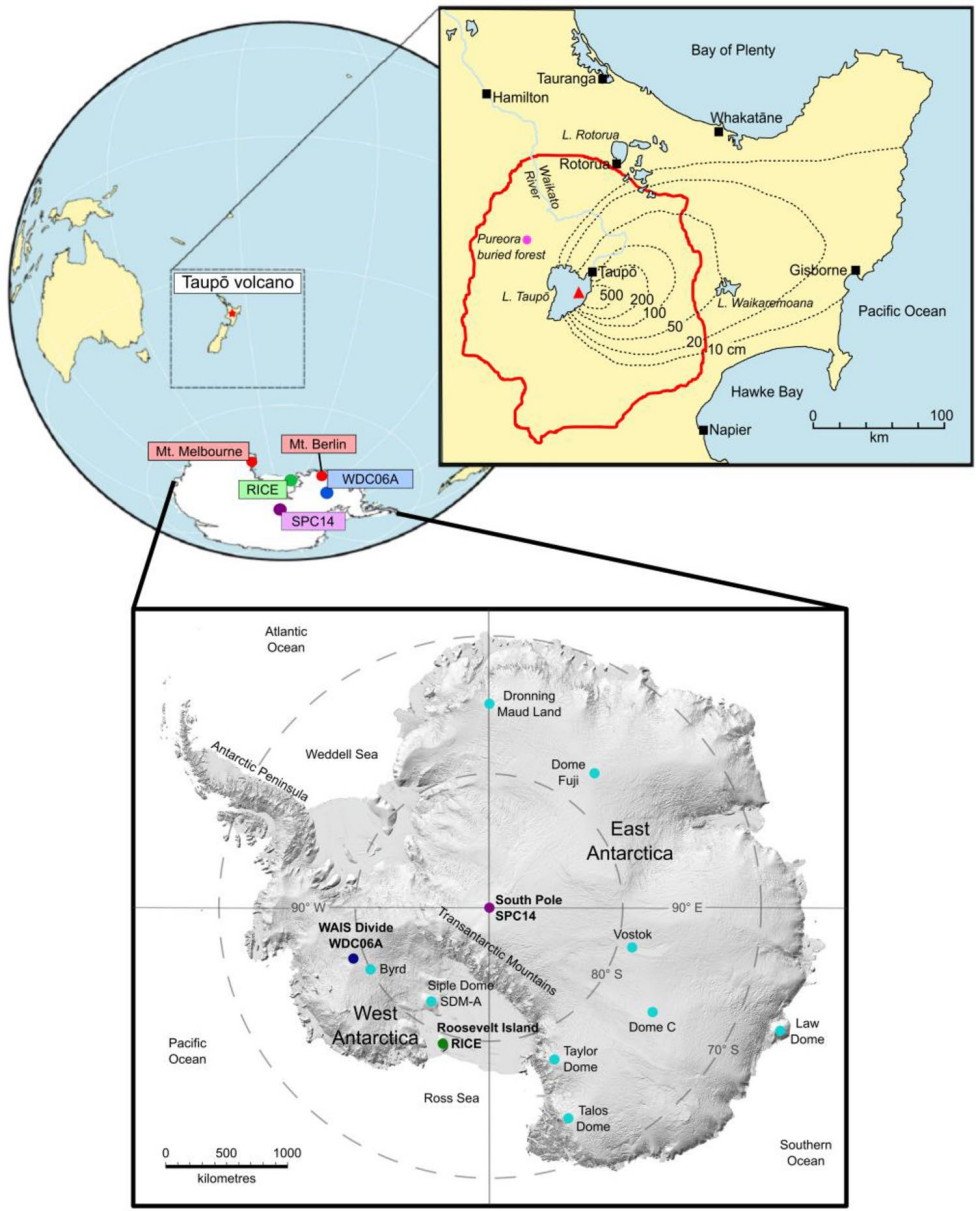
Location of the RICE (green circles), WDC06A (dark blue circles), and SPC14 (purple circles) ice cores, and Mt. Berlin and Mt. Melbourne volcanoes (red circles) in Antarctica relative to the location of Taupō volcano, New Zealand (red star; after Dunbar et al.^13^). Inset map of New Zealand shows the location of the main eruptive vent (red triangle) and Pureora buried forest (pink circle: Clarkson et al.^62^), as well as the outer limit of the Taupō ignimbrite (red line) and tephra fallout isopachs (dashed lines; summed thickness of all phases in cm) from the Taupō eruption (after Wilson and Walker^28^; Wilson^29^). Inset map of Antarctica (after Howat et al.^70^; Johnson et al.^71^) shows the location of the targeted ice cores relative to other Antarctic ice cores (light blue circles). For more information about the characteristics of the selected ice cores refer to Table S1.

Located in the southwest Pacific Ocean, New Zealand hosts two large rhyolitic caldera volcanoes that have been frequently active through the late Quaternary^26,27^ and is one of several potential distal Southern Hemisphere sources for cryptotephra in Antarctic ice core records (e.g., the ~25.5 ka Ōruanui supereruption from Taupō volcano^13^). In particular, the most recent explosive eruption of Taupō volcano (global volcanism program [GVP] volcano number 241070; Fig. 1) at ~1.8 ka (generally known as the ‘Taupō eruption’) was one of the largest and most energetic volcanic eruptions on Earth in the past 5000 years (~105 km^3^ bulk volume^28^). The six-stage eruption sequence lasted between several days to several weeks, producing five physically distinct fall deposits that were widely dispersed by strong south-westerly to westerly winds over a large portion of the North Island, with additional material dispersed over the southwest Pacific Ocean^28^. The eruption culminated with a voluminous (~30 km^3^, bulk volume), extraordinarily energetic pyroclastic flow that devastated an area of *c.* 20,000 km^2^^29^.

As a major time-stratigraphic marker horizon in New Zealand, and a large enough event to have had potentially global impacts, the age of the Taupō eruption has been the subject of marked interest and debate. Initially, the eruption was postulated to have occurred just prior to 186 CE based on historical accounts of atmospheric phenomena from Roman and Chinese sources^30^. This assignment was seemingly confirmed by a volcanic sulfate spike in the 180s CE in the Greenland Ice Sheet Project Two (GISP2) ice core record^31^ but has since been disproven. Matching a ^14^C chronology from trees killed by the eruption at the Pureora buried forest (Fig. 1) to the international radiocarbon calibration curve yielded an eruption date of 232 ± 30 CE (2 SD)^32^. This estimate was refined to 232 ± 10 CE (2 SD) by wiggle-matching new high-precision radiocarbon dates from trees at the same site to a regional calibration curve data set for the Southern Hemisphere^33,34^. This age was disputed, however, by Holdaway et al.^35,36^ who proposed that the reported eruption date was inaccurate due to magmatic carbon contamination and instead occurred decades to two centuries later. Validating the age of the Taupō eruption via other chronological methods, such as presented here, is crucial for multiple stratigraphic frameworks and testing the reliability of existing radiocarbon age data around volcanoes.

Given the magnitude and stratospheric plume heights of the Taupō eruption, it is puzzling as to why this event has been so challenging to detect and validate in Antarctic ice cores. To date, reported volcanic anomalies in ice cores from Greenland and Antarctica linked to the Taupō eruption have been assigned varied ice core ages (e.g., 232 ± 3 CE^6^; 236 ± 3 CE^7^; 236 ± 33 CE^15^ [all at a 95% confidence interval]), but have not yet been verified by the geochemical fingerprinting of insoluble eruptive material (i.e., volcanic glass). Here, we investigate volcanic signals from specific depth intervals in three Antarctic ice cores (Fig. 1**)**, the model ages of which temporally overlap with the Taupō eruption radiocarbon wiggle-match date of 232 ± 10 CE; and report the identification and geochemical characterisation of Taupō-derived glass shards in the Roosevelt Island Climate Evolution (RICE) ice core.

## Results and implications

Samples of the West Antarctic Ice Sheet (WAIS) Divide (WDC06A), South Pole (SPC14), and RICE ice cores (Fig. 1) were chosen for this study based on observations of elevated levels in volcanogenic glaciochemical signals (sulfate, nssS, non-sea-salt conductivity [nss cond^15^]) and increased particle counts over core sections that spanned the reported date of the Taupō eruption^33,34^, using the WD2014^37^, SPC14-02, and RICE17^15^ chronologies (Figs. S1–S3). Particle spikes were often observed down-core from volcanogenic glaciochemical anomalies, consistent with previous studies^10,21,38^. Note that since sampling, the SP19 chronology^39^ has replaced SPC14-02, with new intervals in the time period of interest subsequently identified down-core for further investigation.

**Figure 2:**
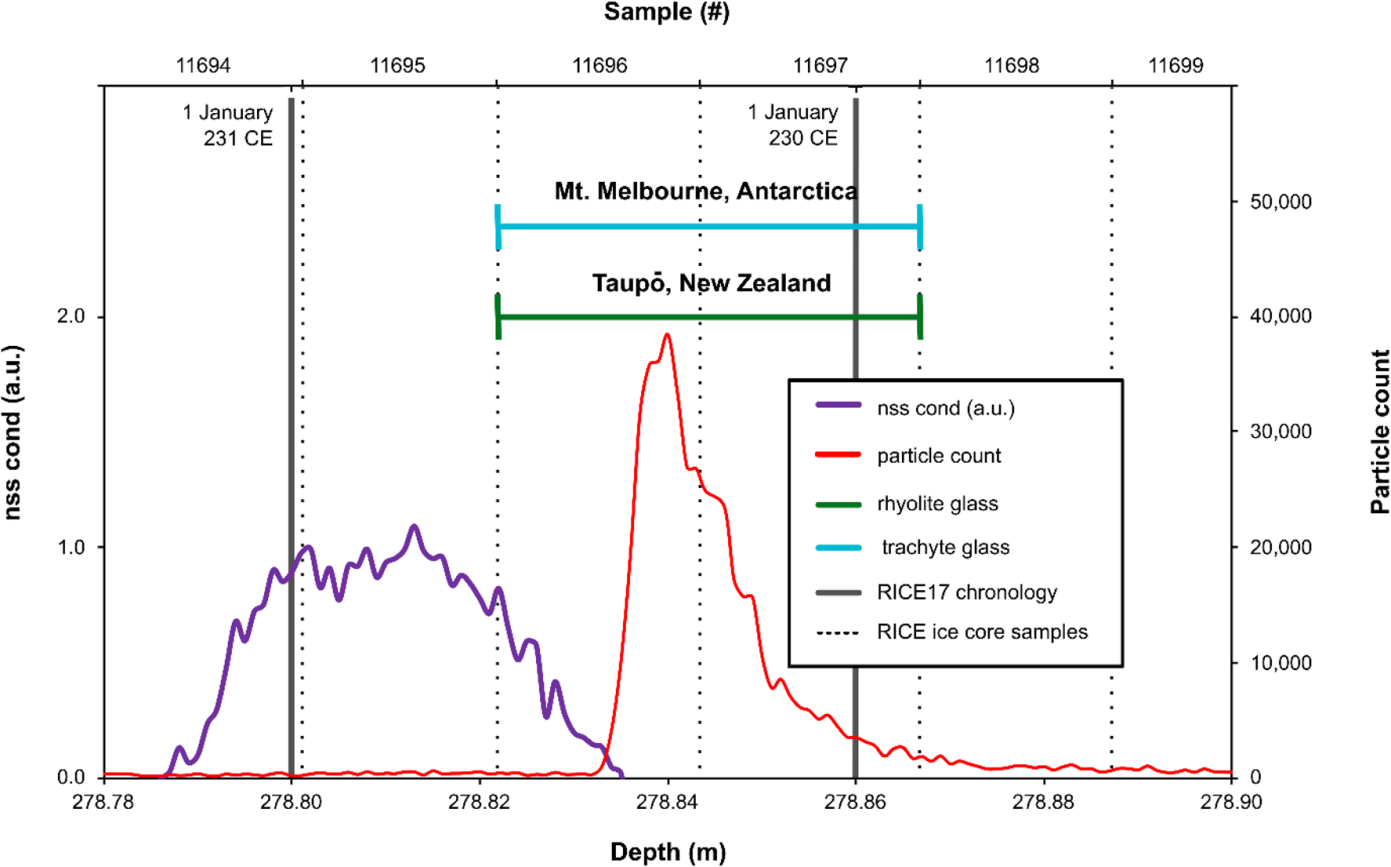
Nss cond (purple) signal and particle counts (red) from the RICE ice core. The nss cond component was obtained by Winstrup et al.^15^ as the conductivity-to-calcium excess (nss conductivity = conductivity – (a*[Ca^2+^] + *b*), with *a* and *b* calculated from linear regression). Populations of Taupō-derived rhyolite (green) and Mt. Melbourne-derived trachyte glass (blue) were identified and analysed in archived samples 11696 and 11697 (dashed lines) of the RICE ice core, spanning 278.822 m to 278.866 m depth, with a RICE17 age of 230 CE^15^. The RICE17 annual layer marks (dark grey lines) were assigned a nominal date of 1 January and were produced based on multiple chemistry series in parallel using the StratiCounter algorithm^15^. Insoluble particles were detected and counted using an expanded version of the Copenhagen CFA system (see Winstrup et al.^15^).

**Figure 3:**
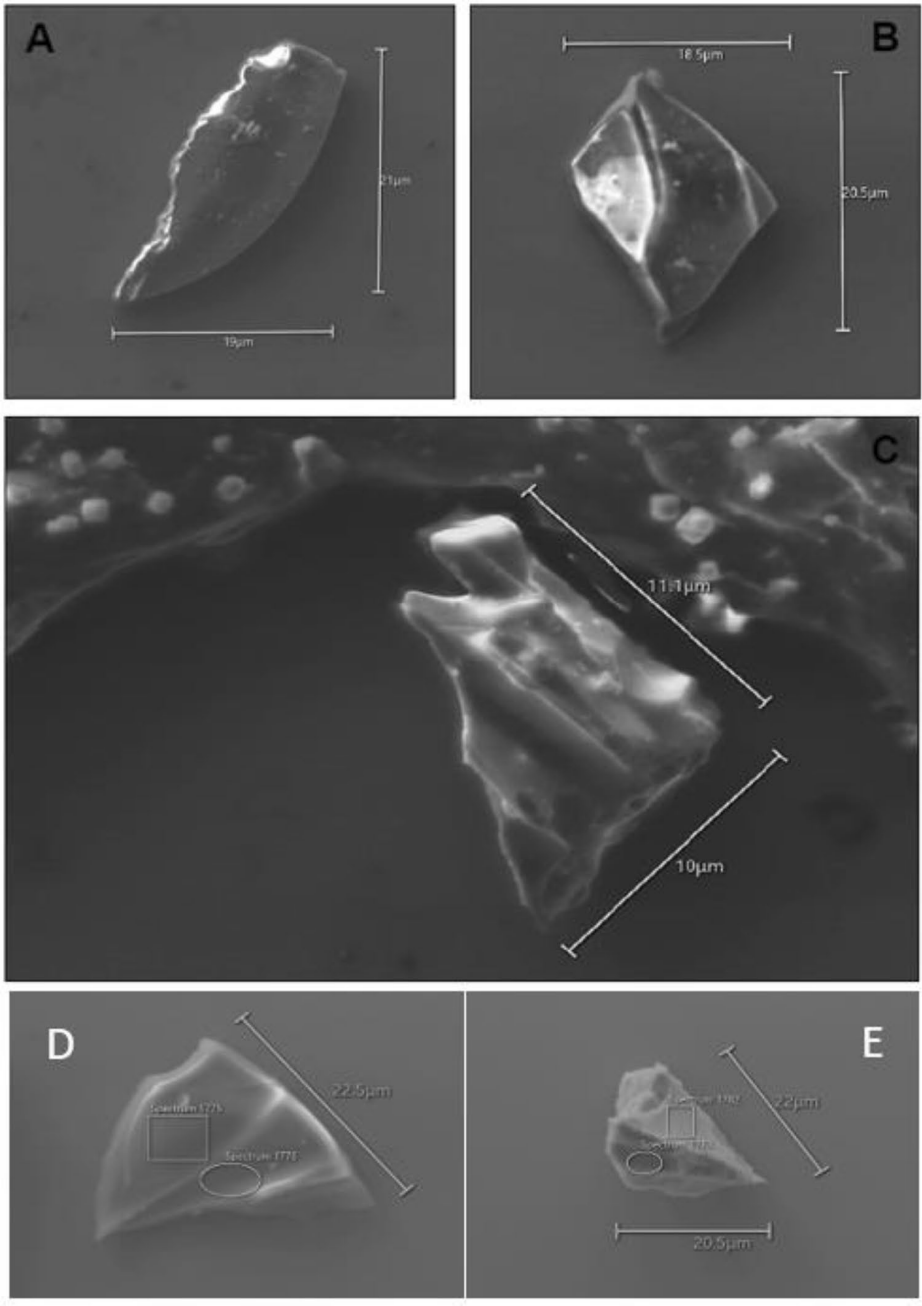
**(A–C)** SEM images of rhyolitic volcanic glass shards isolated from RICE ice core sample 11696 (278.822–278.843 m depth). Note the presence of cubic NaCl grains on and adjacent to the Taupō-derived glass shards.** (D–E)** SEM images of trachytic volcanic glass shards isolated from RICE ice core sample 11697 (278.844–278.866 m depth).

**Figure 4:**
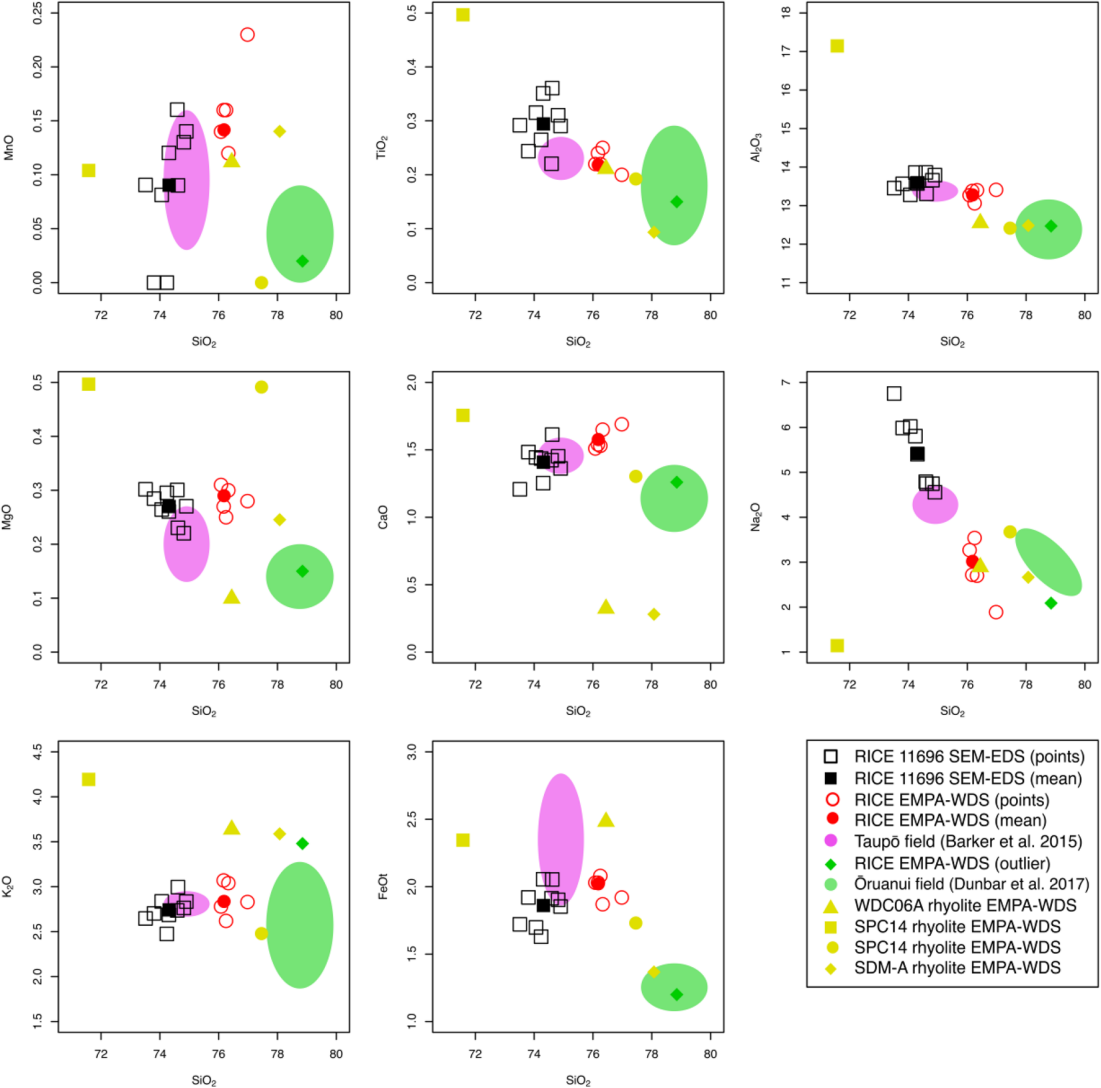
Geochemical compositions of cryptotephra particles analysed from the RICE (black squares and red circles), WDC06A (yellow triangles), SPC14 (yellow squares and circles), and SDM-A (yellow diamonds) ice cores compared to compositional fields for the 232 CE Taupō- (pink ovals: Barker et al.^41^) and ~25.5 ka Ōruanui-derived glass (green ovals: Dunbar et al.^13^). Individual measurements (black open squares) and mean value (black filled squares) from unpolished SEM–EDS of three glass shards identified in RICE ice core sample 11696, distinguished from the individual (red open circles and green filled diamonds) and mean (red filled circles) geochemical measurements obtained from polished EMPA-WDS of six glass shards identified in RICE ice core samples 11696 and 11697.

**Figure 5:**
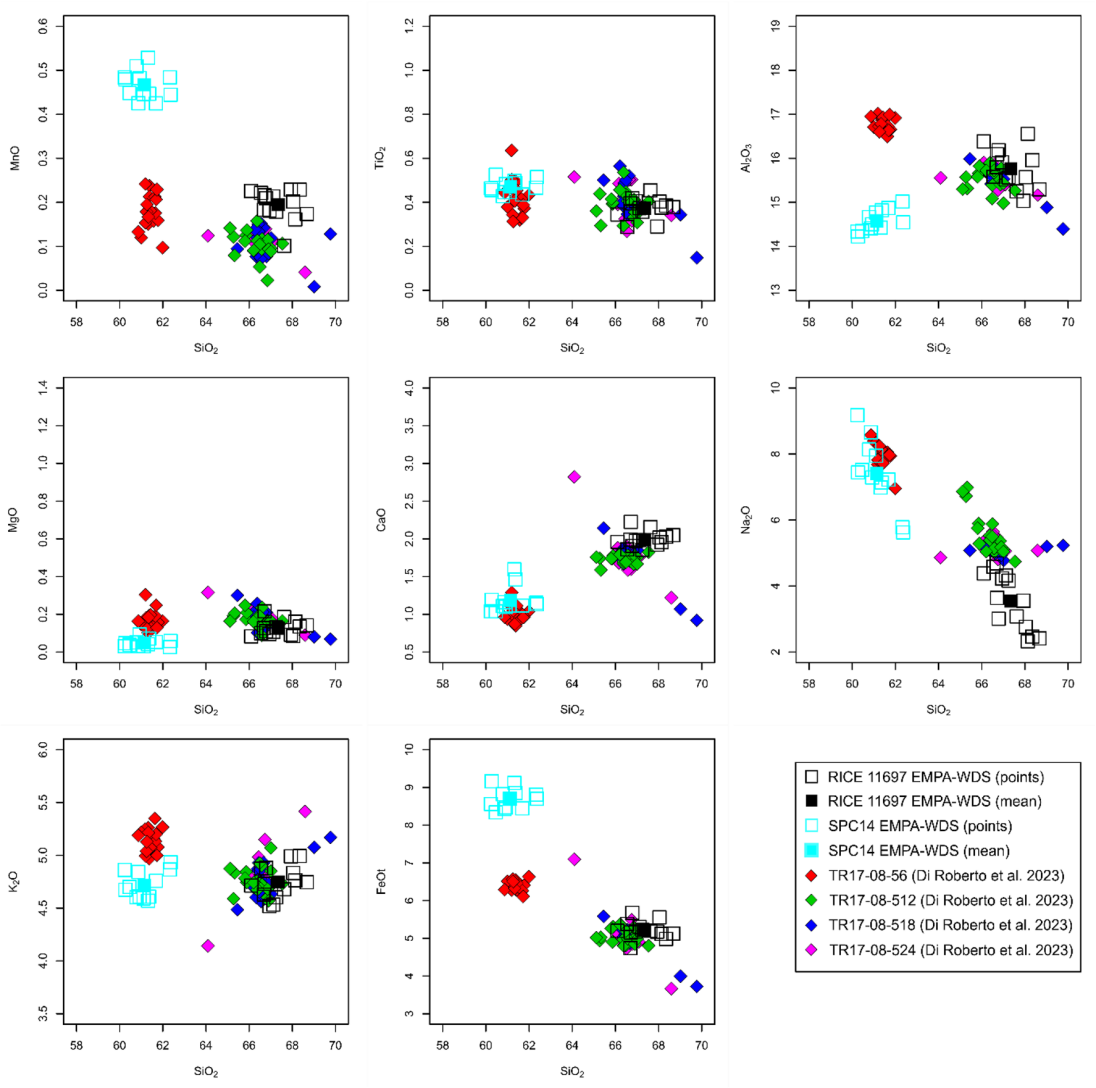
Geochemical compositions of cryptotephra particles analysed from the RICE (black squares) and SPC14 (cyan squares) ice cores compared to four cryptotephra layers identified by Di Roberto et al.^52^ in a marine sediment core collected in the Edisto Inlet, Ross Sea. They include the TR17-08–56 cryptotephra (red diamonds) produced by a historic eruption of Mt. Rittmann at 1254 CE, and three additional cryptotephra layers from Mt. Melbourne at 335 CE (TR17-08–512: green diamonds), 302 CE (TR17-08–518: blue diamonds), and 273 CE (TR17-08–524: pink diamonds)^52^. Note the individual measurements (open squares) and mean value (filled squares) from polished EMPA-WDS of glass shards identified in our samples of the RICE and SPC14 ice cores.

From the three records examined, only one segment in the RICE ice core between 278.822 m and 278.866 m depth (samples 11696 and 11697: Figs. 2, S1), contains a population of rhyolitic volcanic glass shards (Figs. 3A–C, 4). These seven shards are 10–20 µm long, platy to cuspate in shape, some with vesicle walls and others with stretched vesicles with chipped or abraded edges (Figs. 3A–C). They were first analysed in an unpolished state by scanning electron microscopy with energy dispersive spectrometry (SEM–EDS), and subsequently in a polished state by electron probe microanalysis using wavelength dispersive spectrometry (EMPA-WDS). The results show that the shards have a rhyolitic composition (Figs. 3A–C, 4; Supplementary File 1). These rhyolite shards are accompanied by a compositionally distinct population of slightly coarser, zoned trachytic-trachydacitic glass shards 10–25 µm long (Figs. 3D–E, 5) with thicker bubble walls. With the exception of a single rhyolite shard that is compositionally unique (Fig. 4), both glass populations are chemically uniform and imply that there are two distinct sources (Fig. S4). These coincident glass shard populations have an ice core age of 230 ± 32 CE (95% confidence) (RICE17 chronology^15^) and are followed by an elevated nss cond signal (Figs. 2, S1). To evaluate and further refine this age model, we have performed an independent synchronization of selected volcanic tracers between the RICE and WDC06A ice core records, to the high-precision WD2014 chronology^37^ (see Supplementary File 2). The corresponding WD2014 age of the cryptotephra-bearing RICE layer is 230 ± 3 CE (95% confidence). This age is conditional to the correctness of the volcanic synchronization at this depth interval, which is hampered by the low signal-to-noise properties inherent in the RICE volcanic tracers (i.e., acidity, conductivity, and nss cond) due to the strong marine influences at this site^15^. Consequently, we also estimate the age of this layer relative to the well-constrained ages of five major volcanic eruptions (see Supplementary File 2). The volcanic ice core synchronization for these larger eruptions is arguably more robust, and cumulative annual layer counting uncertainties in RICE17 starting from these marker events are well below ± 20 years (95% confidence) (Supplementary File 2). Starting at five different volcanic match-points, the mean resulting age for the cryptotephra-bearing RICE layer is 231 CE ± 16 years (95% confidence). Finally, as the most conservative approach, we only consider the relative RICE17 annual layer counts relative to the Samalas eruption dated to spring/summer 1257 CE, which carries no volcanic synchronization uncertainty because of a common tephra match between WDC06A and RICE at 1252 CE^40^. Reducing the uncertainties in RICE17 for the Samalas layer to ± 0 years gives a revised age of the RICE cryptotephra-bearing layer of 230 ± 19 CE (95% confidence). All three approaches (direct synchronization to the WD2014 chronology^1^; relative layer counting starting from multiple well-dated volcanic marker horizons^2^; and relative layer counting starting from the 1257 CE Samalas eruption^3^) thus converge to an age for this RICE ice core interval of 230 CE with age uncertainties between ± 3 years (conditional to the correctness of the volcanic synchronization) and ± 19 years (95% confidence) (Supplementary File 2).

The geochemical composition of the main rhyolite glass population (with normalized averages of 76.4 wt% SiO_2_, 13.3 wt% Al_2_O_3_, 2.0 wt% FeO, 2.9 wt% K_2_O, and 2.8 wt% Na_2_O measured by EMPA-WDS; Fig. 4, Supplementary File 1) does not match any local Antarctic sources. This rhyolite glass population instead falls closely to the unique compositional field of the 1.8 ka Taupō eruption groundmass glass^41^ (Fig. 4, Supplementary File 1), but with slightly higher SiO_2_. Through examination of geochemical signatures and chronological constraints, the main RICE rhyolite glass population does not correlate geochemically or chronologically with any known Holocene volcanic activity from local Antarctic centres^17,20,42,43^, or other distal Southern Hemisphere sources, including the Austral and Southern Volcanic Zones of the Andes^19,44,45^, or even other New Zealand rhyolite sources^46–49^. No such composition is known to occur in the recent volcanic record from local Antarctic centres^42,50^. The rhyolite shards also display generally higher FeO contents than reported in glasses from broadly contemporaneous activity at South and Central American volcanoes (Supplementary File 1). Furthermore, the main RICE rhyolite glass population is also compositionally distinguishable for selected major elements even when considering younger powerfully explosive rhyolite eruptions, such as the 431 CE Tierra Blanca Joven eruption, which is >2.5 wt% higher in SiO_2_ than our englacial rhyolite glass (Supplementary File 1). Aside from the Taupō eruption, no other known events have the geochemical characteristics or eruption age estimates compatible with those of the main RICE rhyolite glass population. Furthermore, the solitary compositionally distinct rhyolitic glass shard found with the 1.8 ka Taupō eruption shards (Fig. 4) is indistinguishable within analytical uncertainty from the Ōruanui-derived cryptotephra identified by Dunbar et al.^13^ in the WDC06A ice core (Supplementary File 1). The presence in the RICE ice core sample of such a shard with geochemical characteristics indistinguishable within error to the published narrow compositional field of Ōruanui-derived glass^13,51^ provides a unique and definitive second fingerprint for the Taupō volcano source.

Based on major element composition the trachyte glass shard population (with normalized averages of 67.4 wt% SiO_2_, 15.8 wt% Al_2_O_3_, 5.2 wt% FeO, 4.8 wt% K_2_O, and 3.5 wt% Na_2_O: Fig. 5, Supplementary File 1) from the RICE ice core (Figs. 2, S1) has geochemical affinity to cryptotephra particles^52^ derived from past explosive eruptions of Mt. Melbourne, Northern Victoria Land (GVP volcano number 390015: 74˚20′60″ S, 164˚41′60″ E; 2,732 m asl^53^; Fig. 1) suggesting a likely local source and correlation to an eruption previously unidentified in the Antarctic ice cores (Fig. 5). The RICE englacial cryptotephra is, however, slightly more silicic and enriched in CaO and MnO compared to the most recent deposits found on Mt. Melbourne (Supplementary File 1), which likely originate from Strombolian to sub-Plinian/Plinian-scale explosive eruptions^53,54^. The trachytic-rhyolitic pumice fall deposits that compose the crater rim and are extensively dispersed around Mt. Melbourne have been difficult to date because of their young age^53,54^. We instead geochemically correlate our englacial cryptotephra with three cryptotephra layers reported by Di Roberto et al.^52^ that are derived from younger, previously unknown, explosive eruptions of Mt. Melbourne, emplaced between the 3rd and 4th centuries CE (Fig. 5, Supplementary File 1).

We observe slight variations in glass compositions between the two measurement techniques (SEM–EDS versus EMPA-WDS) and within populations measured using the same instrument. The SEM–EDS population variability, notably in K_2_O and CaO can be explained by the particles being unpolished^24^ and having a rough surface geometry^55^. Despite a high standard deviation for some minor element oxides, however, unpolished SEM–EDS measurements have been demonstrated to be reliable for the purposes of geochemical characterisation and (crypto)tephra comparisons^22^. However, there are measurable Na_2_O variations between SEM–EDS and EMPA-WDS data for the rhyolitic and trachytic glass shards (Figs. 4 and 5). The EMPA-WDS results show higher SiO_2_ wt% and lower Na_2_O wt% compared to the SEM–EDS measurements of the same particles. This contrast is inferred to reflect Na loss during initial SEM–EDS analysis due to the unavoidable use of small beam sizes required because of the shapes and small grain sizes of the shards, further compounded by multiple analyses of the same particles^56^. For the RICE rhyolite glass population, SEM–EDS analysis on unpolished glass surfaces yielded anomalously high Na_2_O wt% (Fig. 4, Supplementary File 1), which likely resulted from the presence of adhering NaCl crystals (Fig. 3A–C). The relationship between Na_2_O wt% and SiO_2_ wt% for multiple analyses of individual glass shards and between particles (Fig. 4) appears linear (reflecting the 100% summing) and Na loss or gain can therefore be estimated and accounted for.

We did not find a definitive horizon of volcanic glass related to the Taupō eruption in any of the other ice core samples we examined. Only one rhyolite glass shard (~10 µm), which is close in composition to Taupō-derived glass except for significantly lower CaO wt% (Fig. 4, Supplementary File 1), was identified in an interval of the WDC06A ice core between 431.35 and 431.65 m depth (Fig. S2), corresponding to an age of 231 ± 3 CE (WD2014 chronology^37^). There was also a trachyte population of volcanic glass identified in the SPC14 ice core at 176.54 m to 176.64 m depth (Fig. S3), which has a geochemical affinity to other englacial cryptotephra derived from Mt. Berlin (GVP volcano number 390022: 76˚02′60″ S, 136˚00′00″ W; 3,478 m asl; Fig. 1) (Fig. 5, Supplementary File 1). This volcano has been one of the most active ash-producers in Antarctica over the past 500,000 years and is a major source for (crypto)tephra layers found in East and West Antarctic ice sheets^17,20^.

## Discussion

Detection of glaciochemical and physical traces of volcanic eruptions in polar ice core records helps to construct, constrain, and correlate chronologies^15,37,39^. Sulfur anomalies are often attributed to well-known volcanic eruptions (particularly during historic times), not only offering chronological constraints on annual layer counting but also providing insights into volcanogenic forcing of climate^9^. Identification of volcanic glass in ice cores provides a geochemical fingerprint to determine the source volcano, and is particularly important at depths where annual layer counting becomes challenging, and ice age uncertainties greatly increase^13,15,20,42^. In turn, well-defined ice core chronologies, particularly in ice <2000 years old, provide a means to verify eruption age if an eruptive source can be uniquely identified^57^.

Our analysis of rhyolite glass particles from RICE strongly imply that Taupō is the source volcano, with major element compositions that are indistinguishable from groundmass glass previously analysed from juvenile Taupō pumice clasts from all phases of the eruption^41^ (Fig. 4). In addition, the presence of an individual rhyolite shard compositionally indistinguishable from the remarkably uniform geochemistry of Ōruanui-derived glass^13,51^ acts as a diagnostic double fingerprint for Taupō volcano and precludes any other potential source. The presence of Ōruanui-derived glass is not surprising if the high plume that delivered the shards to Antarctica was that associated with the final exceptionally energetic outburst that generated the Taupō ignimbrite. This phase of the eruption involved evacuation of >2 km^3^ of millimeter- to meter-sized fragments of country-rock material around the vent^29^, probably accompanying caldera collapse. It is therefore inevitable that additional volumes of ash-grade material from the earlier Ōruanui caldera fill would have been excavated and incorporated into the exceptionally powerful co-ignimbrite plume that would have penetrated the stratosphere^28,29^. A similar bimodality of volcanic glass compositions is reported from the quantitative geochemical analysis of proximal tephra deposits from the 1.8 ka Taupō eruption^58^ and again matches published compositions of glass from juvenile materials in the Taupō eruption itself^41^ and the Ōruanui^51^. We also suggest that the coevality of the Taupō and Mt. Melbourne eruption traces could be used as a new, precisely-dated, independent, and reliable time-stratigraphic marker horizon for the developing Antarctic tephrochronological framework, and potentially linked to circum-Antarctic marine sediment records^52^. Our discovery of Mt. Melbourne-derived cryptotephra in the RICE ice core (Fig. S1) supports the conclusion of Di Roberto et al.^52^ that the Mt. Melbourne volcanic complex was highly active in historical times and, in the case of the Taupō eruption, may obscure records of distal cryptotephra.

The current age estimate of the Taupō eruption is 232 ± 10 CE (2 SD), based on wiggle-matching high-precision radiocarbon dates from trees buried and preserved by the climactic pyroclastic flow^33,34^. However, this age estimate was challenged by Holdaway et al.^35,36^ who proposed that ^14^C measurements on material from close to the volcano may be contaminated by magmatic infinite-age carbon introduced into the growing trees from groundwater. Holdaway et al.^35,36^ instead asserted that the Taupō eruption occurred at an unknown date, decades to two centuries later, bringing into question the use of the Taupō Tephra as a reliable time-stratigraphic marker. Their proposal has significant implications for both the timing of the Taupō eruption and also for other proximally-dated, unobserved, eruptions such as the Minoan eruption of Thera^59^ (Santorini) and the 1314 ± 12 CE (2 SD) Kaharoa eruption^60^ (New Zealand).

The current 232 ± 10 CE radiocarbon age estimate for the Taupō eruption^33,34^ is here validated by geochemical fingerprinting of Taupō-derived volcanic glass in the RICE ice core. In addition, further examination of the record for the RICE ice core, including the section containing the Taupō-derived shards, permits a more precise estimate to be made of the age of the ice at this time. For this section (between 42.34 and 343.70 m; 1885 CE–700 BCE), the RICE17 age model is an independent annual-layer-counted chronology^15^ that was augmented using the StratiCounter layer-counting algorithm^61^ to interpret annual signals based on the full suite of continuous-flow analysis (CFA) records (black carbon, acidity, insoluble dust particles [42.3–129 m depth], Ca^2+^, and conductivity) in parallel, as necessitated by changing properties and availability of data with depth. The RICE17 annual-layer marks (grey lines; Fig. 2) were assigned a nominal date of 1 January, following the identification of summer signals^15^. Through synchronization of the RICE and WDC06A ice core records we have independently confirmed the date of the Taupō eruption to 230 ± 19 CE (95% confidence) (Supplementary File 2), which allows us to firmly refute propositions of a potential age bias arising from contamination by magmatic indeterminate-age carbon^35,36^.

The location of the particle peak containing Taupō- and Mt. Melbourne-derived volcanic glass in the RICE ice core starts near the beginning of the year (as defined by StratiCounter) and is at a maximum within a region corresponding to late summer/early autumn. This specific time of year is remarkably consistent with the independently proposed timing (late March to early April in a typical year) for the climactic phase of the Taupō eruption from records in the Pureora buried forest (Fig. 1) on the basis of fruit and seeds, and the lack of an outer latewood ring on buried logs^62,63^. We therefore conclude that the existing radiocarbon age estimate of Hogg et al.^33,34^ for the Taupō eruption is robust and that the ice core record supports the final phase of the eruption occurring in the late summer/early autumn of 230 ± 19 CE.

The deposition of glass particles from the 1.8 ka Taupō eruption in Antarctica ~5000 km away was likely related to the exceptionally powerful eruption plume associated with the climactic emplacement of the Taupo ignimbrite^29^. Injection into the stratosphere and subsequent atmospheric circulation of volcanic aerosols and particles over polar surfaces^13^ via the Antarctic Polar Vortex suggests that this valuable time-stratigraphic marker should be present in more Antarctic ice cores. Travel distances for eruptive clouds depositing (crypto)tephra have been reported to exceed 7,000 km along dominant wind dispersal directions for moderate^10^, and large explosive volcanic eruptions^64^, including the Ōruanui event^13^. In the case of the Taupō eruption, the predominant dispersal path of ash over the Pacific Ocean to the east of the vent is consistent with the prevailing tropospheric winds and strengthening westerly flow in the stratosphere during early autumn^65^. Neff and Bertler^66^ also demonstrated that there are several potential synoptic tropospheric transport pathways for dust particles to travel from New Zealand to Antarctica. We therefore infer that particles from Taupō may have been feasibly transported to, deposited, and will be recorded in other Antarctic ice core records (i.e., WDC06A).

A major challenge with precisely locating the Taupō event is the subtle nature of any associated volcanogenic glaciochemical signals in available ice core records. Of the three ice cores investigated here, only the RICE ice core contains a population of rhyolite glass that can be confidently linked to the Taupō eruption. With its coastal location and low altitude, the site of the RICE ice core receives a significant seasonal influx of sulfur-bearing compounds from biological oceanic sources, with peak values of up to 200 ppb of nssS^15^. The seasonal peaks in biogenic nssS are on the same order of magnitude as the expected sulfate deposition from large volcanic eruptions, inhibiting identification of these events using traditional volcanic ice core tracers. Instead, detection of volcanic horizons in the RICE ice core relies on direct measurements of total acidity and estimated nss cond^15^. For this study, we selected intervals in the RICE ice core based on particle counts and nss cond^15^ and found Taupō glass in a section of core that would usually be overlooked for the subtle nature of the eruption signature. While sulfate data are not available for the interval of interest in the RICE ice core, the likely corresponding interval of the WDC06A ice core (Fig. S5) contains a broad nssS anomaly, which has previously been attributed to the Taupō eruption by Sigl et al.^6^ at ~ 231/232 CE and later revised to 236 ± 3 CE (95% confidence)^7^. The glass particles are either absent from this section of the core, or they are present in bracketing ice not selected for melting, highlighting the challenges of pinpointing this event. The stratospheric sulfur injection calculated for this attributed sulfate signal using a total of 12 ice cores (10 from Antarctica and 2 from Greenland) of 5.8 ± 1.2 Tg of sulfur^67^ is comparable to that calculated using petrological techniques of ~6.7 Tg of sulfur^68^. This is a relatively low sulfur emission given the size of the eruption and reflects the lower concentration of sulfur in the rhyolite magma when compared to other smaller volume, but sulfur-rich events^68^. Although while during the Taupō event, the maximum values of sulfate in each annual cycle from the WDC06A and NEEM S1 ice cores were only moderately enhanced compared to other volcanic signals, the annual minimum values of sulfur exceeded the natural background for up to 7 consecutive years^6^. This moderate but longer-lasting flux of volcanic sulfur has been suggested to result from the high stratospheric plume and subsequent size-dependent fallout of volcanic aerosols from the stratosphere. These findings highlight that other large volume eruptions of sulfur-poor magmas may be missed in ice core records and that nssS anomalies are not necessarily indicative of eruption size but rather plume height and aerosol transport pathways^5^.

In conclusion, we have identified rhyolite glass shards from the 1.8 ka Taupō eruption in Antarctic ice for the first time, providing an eruption date estimate of 230 ± 19 CE (95% confidence). This date is consistent with the radiocarbon wiggle-match date estimate of 232 ± 10 CE (2 SD) from material buried by the eruption deposits^33,34^, and with previous inferences that the eruption occurred in late summer/early autumn (late March to early April in a typical year^62,63^). An accurate age estimate offers a new time period within which possible global impacts (i.e., atmospheric effects^30^ or volcanogenic cooling^6,7,9^) could be sought, particularly in palaeoclimate records from the Northern Hemisphere. Six of the seven rhyolite glass shards analysed match published glass chemistry of the Taupō eruption products, while a seventh shard matches the composition of the ~25.5 ka Ōruanui eruption^51^, also from Taupō volcano and previously identified in the WDC06A ice core by Dunbar et al.^13^. The combined presence of these two distinctive compositions thus absolutely pins the horizon in which the shards were found to a Taupō volcano source. In this instance, eruptive particles were not found in association with glaciochemical proxies for volcanism previously attributed to the Taupō eruption in the RICE ice core^15^, providing an important opportunity to improve the accuracy of the RICE17 chronology. Furthermore, while populations of Taupō-derived cryptotephra were not located in the WDC06A and SPC14 ice core samples examined in this study, promising chronological links may be developed following identification and correlation to the temporally coincident Mt. Melbourne cryptotephra. The relationship between these two geochemically distinct eruptive deposits in the RICE ice core may lead to recognition of eruptive material from the Taupō eruption in other Antarctic ice cores. Both eruptions combined provide a valuable, precisely-dated chronostratigraphic marker for the developing Antarctic tephrochronological framework.

## Methods

### Preparation of samples for imaging and geochemical analysis

Large volume samples (>10 ml) of the WDC06A and SPC14 ice cores were cut at the National Science Foundation Ice Core Facility (NSF-ICF; Denver, Colorado, USA) and processed at New Mexico Tech (NMT). Small volume filtered meltwater samples (<10 ml) from the outer section of the RICE ice core were obtained from the New Zealand National Ice Core Research Facility (Wellington, New Zealand). To account for volumetric differences between samples of the WDC06A, SPC14, and RICE ice cores, we modified the sample preparation procedures developed by Dunbar et al.^69^ and Iverson et al.^22^. Large and small volume samples were prepared differently to maximise particle extraction, summarised in a sample preparation flow chart (Fig. S6).

The WDC06A and SPC14 ice core samples were prepared at NMT using the large volume procedures (Fig. S6A–D). Each sample was melted at room temperature and filtered through a 0.2 µm nucleopore polycarbonate membrane. A folded piece of polyimide tape was then wiped across the upper surface of the filter to pick up particles deposited during the filtering process, concentrating them along the fold. The tape was then flattened, pressed onto a 25-mm diameter aluminium disk, which had been coated with a thin layer of light adhesive (Mikrostik™), and peeled back leaving some of the particles on both the disk and tape (Fig. S6A). The disk was then attached, with double-sided carbon tape to a cylindrical aluminium specimen stub for uncoated SEM–EDS characterization. Up to four samples could be adhered to the same disk. The disks were manufactured by drilling and dremeling aluminium hard disk platters (HDPs) removed from used computer hard drives. They provide a conductive, ultra-flat surface that Iverson et al.^22^ found maximises particle recovery, improves the polishing quality of exposed grains, and minimises any material loss associated with over-polishing. After the samples were imaged and analysed, a tape dam was adhered around the disk and filled with EpoThin epoxy (Fig. S6B). Following curing, the EpoThin round was separated from the disk using a razor blade and, where necessary, prepared for EMPA-WDS (Fig. S6D). Once cleaned the disk could also be reused.

Particles remaining on the polyimide tape were mounted in epoxy by affixing the tape to the bottom of a polished (with 30 µm and 15 µm grits) and 4-hole epoxy round, which was then backfilled with low viscosity Spur 4-part epoxy and cured in an oven at 80 °C for 4 hr (Fig. S6C). To ensure that the tape did not wrinkle during curing, a lead weight was placed on top of the mount. By using the same epoxy for the round and the samples, the surface of the backfilled holes was found to remain flatter, making the mounts easier to polish and reducing the potential for material loss by over-polishing. Mounts prepared using either of these procedures were then polished with diamond powder suspended in deionised water (Fig. S6D). Typically, samples were polished for 2 min with 6 µm and for another 2 min with 1 µm grits. Polishing times vary depending on the sample and it was considered important to check the sample during polishing at 30 sec intervals on a petrographic microscope using both transmitted and reflected light to ensure that the grains had been exposed then to preclude them from being polished away. Once polished, the sample was carbon coated and made ready for quantitative WDS analysis. The parallel sample preparation method allowed for multiple microanalytical methods to be performed on a single sample and provided a backup in case one of the two preparation procedures was unsuccessful.

The RICE ice core samples were prepared at the New Zealand National Ice Core Research Facility using the small volume procedures, which were similar to those above and developed by modifying the methods of Iverson et al.^22^ for ‘cryptotephra’ samples (Fig. S6E). Samples were melted at room temperature in 15 ml polycarbonate vials and then centrifuged at 3,000 rpm for 5 min. Liquid from the bottom of the test tube was pipetted onto the surface of an aluminium disk, which was then heated to 75 °C on a hot plate. Three to nine 40 µl aliquots of particle-bearing liquid were evaporated on the hot plate, concentrating the particles on the upper surface of the HDP. This process was observed under a stereoscopic microscope and the outer boundaries of each aliquot were outlined with permanent marker. Once cooled, the disk was attached to a specimen stub with double-sided carbon tape and the sample material concentrated on the upper surface of the disk was ready for SEM–EDS without carbon coating. If further quantitative analysis was required, a tape dam was adhered around the disk, which was then filled with EpoThin epoxy, cured and polished following the same process as described above for large volume samples (Fig. S6B, D). The remaining meltwater was then filtered through a 0.2 µm filter, which was archived and can be prepared for future imaging and geochemical analysis following the parallel preparation procedures outlined above for filtered large volume samples (Fig. S6A–D).

### Analytical methods

Unpolished samples were imaged and characterised by SEM–EDS. Images and geochemical measurements, particularly of small grains (<10 µm in diameter), were obtained at NMT with a JEOL JSM-IT700HR SEM equipped with an Oxford Instruments X-Max^N^ EDS detector, respectively, using an accelerating potential of 15 kV and a probe current of 75 nA. Due to the low number and small size of analysable particles, SEM–EDS analysis of unpolished samples was critical to locate and document the geochemical signature, size, and shape of microscopic volcanic glass shards.

Quantitative geochemical analyses of polished samples were performed by EMPA-WDS on a 4 WDS spectrometer Cameca SX-100 Electron Probe Microanalyzer at NMT using a 15 kV accelerating potential and 10 nA probe current, with beam diameters of 20, 15 or 10 µm, depending on particle size. In some instances, beam sizes slightly larger than the glass particles were used resulting in low analytical totals due to the overlap on epoxy following the broad beam overlap method of Iverson et al.^22^. All analyses were normalized to 100 wt% to allow comparison of all particles. This technique was effective at minimising the unavoidable Na loss associated with analysing small particles and did not adversely affect analytical accuracy; however, the precision for a set of points may decrease^22^. A set of secondary standards, including rhyolitic glass VG568, were analysed during analytical sessions to monitor accuracy and precision of both unpolished SEM–EDS and polished EMPA-WDS analyses.

### Supplementary Information

Supplementary Information 1.

Supplementary Information 2.

## Data Availability

All data generated or analysed during this study are included in this published article (and its supplementary files).

## References

[1] Jouzel J (2007). Orbital and millennial Antarctic climatic variability over the past 800,000 years. Science.

[2] Legrand M, Mayewski P (1997). Glaciochemistry of polar ice cores: A review. Rev. Geophys..

[3] Koffman BG, Kreutz KJ, Kurbatov AV, Dunbar NW (2013). Impact of known local and tropical volcanic eruptions of the past millennium on the WAIS divide microparticle record. Geophys. Res. Lett..

[4] Cole-Dai J (2021). Comprehensive record of volcanic eruptions in the Holocene (11,000 years) from the WAIS Divide, Antarctica ice core. J. Geophys. Res. Atmos..

[5] Lin J (2022). Magnitude, frequency and climate forcing of global volcanism during the last glacial period as seen in Greenland and Antarctic ice cores (60–9 ka). Clim. Past..

[6] Sigl M (2013). A new bipolar ice core record of volcanism from WAIS Divide and NEEM and implications for climate forcing of the last 2000 years. J. Geophys. Res. Atmos..

[7] Sigl M (2015). Timing and climate forcing of volcanic eruptions for the past 2500 years. Nature.

[8] Plunkett G (2022). No evidence for tephra in Greenland from the historic eruption of Vesuvius in 79 CE: Implications for geochronology and paleoclimatology. Clim. Past.

[9] Sigl M, Toohey M, McConnell JR, Cole-Dai J, Severi M (2022). Volcanic stratospheric sulfur injections and aerosol optical depth during the Holocene (past 11 500 years) from a bipolar ice-core array. Earth Syst. Sci. Data.

[10] Jensen BJL (2014). Transatlantic distribution of the Alaskan White River Ash. Geology.

[11] Marshall LR (2022). Volcanic effects on climate: Recent advances and future avenues. Bull. Volcanol..

[12] Lowe DJ (2011). Tephrochronology and its application: A review. Quat. Geochronol..

[13] Dunbar NW (2017). New Zealand supereruption provides time marker for the last glacial maximum in Antarctica. Sci. Rep..

[14] Cook E, Davies SM, Guðmundsdóttir ER, Abbott PM, Pearce NJG (2018). First identification and characterization of Borrobol-type tephra in the Greenland ice cores: New deposits and improved age estimates. J. Quat. Sci..

[15] Winstrup M (2019). A 2700-year annual timescale and accumulation history for an ice core from Roosevelt Island, West Antarctica. Clim. Past.

[16] Lee JE (2020). An 83 000-year-old ice core from Roosevelt Island, Ross Sea, Antarctica. Clim. Past.

[17] Dunbar NW (2021). Active volcanoes in Marie Byrd Land. Geol. Soc. Lond. Mem..

[18] Palais JM, Germani MS, Zielinski GA (1992). Inter-hemispheric transport of volcanic ash from a 1259 A.D. volcanic eruption to the Greenland and Antarctic ice sheets. Geophys. Res. Lett..

[19] Narcisi B, Petit JR, Chappellaz JA (2010). A 70 ka record of explosive eruptions from the TALDICE ice core (Talos Dome, East Antarctic Plateau). J. Quat. Sci..

[20] Dunbar NW, Kurbatov AV (2011). Tephrochronology of the siple dome ice core, West Antarctica: Correlations and sources. Quat. Sci. Rev..

[21] Koffman BG (2017). Rapid transport of ash and sulfate from the 2011 Puyehue-Cordón Caulle (Chile) eruption to West Antarctica. J. Geophys. Res. Atmos..

[22] Iverson NA, Kalteyer D, Dunbar NW, Kurbatov A, Yates M (2017). Advancements and best practices for analysis and correlation of tephra and cryptotephra in ice. Quat. Geochronol..

[23] Plunkett G (2020). Smoking guns and volcanic ash: the importance of sparse tephras in Greenland ice cores. Polar Res..

[24] Hartman LH (2019). Volcanic glass properties from 1459 CE volcanic event in South Pole ice core dismiss Kuwae caldera as a potential source. Sci. Rep..

[25] Abbott PM (2021). Cryptotephra from the Icelandic Veiðivötn 1477 CE eruption in a Greenland ice core: confirming the dating of volcanic events in the 1450s CE and assessing the eruption's climatic impact. Clim. Past.

[26] Nairn IA (2002). Geology of the Okataina Volcanic Centre, scale 1:50000. Institute of Geological & Nuclear Sciences Geological Map 25.

[27] Barker SJ (2021). Taupō: an overview of New Zealand’s youngest supervolcano. N. Z. J. Geol. Geophys..

[28] Wilson CJN, Walker GPL (1985). The Taupo eruption, New Zealand I. general aspects. Philos. Trans. R Soc. Lond. Ser. A Math. Phys. Sci..

[29] Wilson CJN (1985). The Taupo eruption New Zealand II. The Taupo ignimbrite. Philos. Trans. R Soc. Lond. Ser. A Math. Phys. Sci..

[30] Wilson CJN, Ambraseys NN, Bradley J, Walker GPL (1980). A new date for the Taupo eruption, New Zealand. Nature.

[31] Zielinski GA (1994). Record of volcanism since 7000 B.C. from the GISP2 Greenland ice core and implications for the volcano-climate system. Science.

[32] Sparks RJ (1995). ^14^C calibration in the Southern Hemisphere and the date of the last Taupo eruption: Evidence from tree-ring sequences. Radiocarbon.

[33] Hogg A, Lowe DJ, Palmer J, Boswijk G, Ramsey CB (2012). Revised calendar date for the Taupo eruption derived by ^14^C wiggle-matching using a New Zealand kauri ^14^C calibration data set. Holocene.

[34] Hogg AG (2019). Wiggle-match radiocarbon dating of the Taupo eruption. Nat. Commun..

[35] Holdaway RN, Duffy B, Kennedy B (2018). Evidence for magmatic carbon bias in ^14^C dating of the Taupo and other major eruptions. Nat. Commun..

[36] Holdaway RN, Duffy B, Kennedy B (2019). Reply to ‘Wiggle-match radiocarbon dating of the Taupo eruption’. Nat. Commun..

[37] Sigl M (2016). The WAIS divide deep ice core WD2014 chronology–part 2: Annual-layer counting (0–31 ka BP). Clim. Past.

[38] Plunkett G, Sigl M, McConnell JR, Pilcher JR, Chellman NJ (2023). The significance of volcanic ash in Greenland ice cores during the common Era. Quat. Sci. Rev..

[39] Winski DA (2019). The SP19 chronology for the South Pole ice core–part 1: Volcanic matching and annual layer counting. Clim. Past.

[40] Lee MJ, Kyle PR, Iverson NA, Lee JI, Han Y (2019). Rittmann volcano, Antarctica as the source of a widespread 1252 ± 2 CE tephra layer in Antarctica ice. Earth Planet. Sci. Lett..

[41] Barker SJ, Wilson CJN, Allan ASR, Schipper CI (2015). Fine-scale temporal recovery, reconstruction and evolution of a post-supereruption magmatic system. Contrib. Miner. Petrol..

[42] Narcisi B, Petit JR, Delmonte B, Scarchilli C, Stenni B (2012). A 16,000-yr tephra framework for the Antarctic ice sheet: a contribution from the new Talos Dome core. Quat. Sci. Rev..

[43] Narcisi B, Petit JR (2021). Englacial tephras of East Antarctica. Geol. Soc. Lond. Mem..

[44] Stern CR (2008). Holocene tephrochronology record of large explosive eruptions in the southernmost Patagonian Andes. Bull. Volcanol..

[45] Smith RE (2019). Refining the Late Quaternary tephrochronology for southern South America using the Laguna Potrok Aike sedimentary record. Quat. Sci. Rev..

[46] Stokes S, Lowe DJ (1988). Discriminant function analysis of late Quaternary tephras from five volcanoes in New Zealand using glass shard major element chemistry. Quat. Res..

[47] Smith VC, Shane P, Nairn IA (2005). Trends in rhyolite geochemistry, mineralogy, and magma storage during the last 50 kyr at Okataina and Taupo volcanic centres, Taupo Volcanic Zone, New Zealand. J. Volcanol. Geotherm. Res..

[48] Lowe DJ, Blaauw M, Hogg AG, Newnham RM (2013). Ages of 24 widespread tephras erupted since 30,000 years ago in New Zealand, with re-evaluation of the timing and palaeoclimatic implications of the Lateglacial cool episode recorded at Kaipo bog. Quat. Sci. Rev..

[49] Hopkins JL, Lowe DJ, Horrocks JL (2021). Tephrochronology in Aotearoa New Zealand. N. Z. J. Geol. Geophys..

[50] Narcisi B, Petit JR, Delmonte B, Batanova V, Savarino J (2019). Multiple sources for tephra from AD 1259 volcanic signal in Antarctic ice cores. Quat. Sci. Rev..

[51] Allan ASR (2017). A cascade of magmatic events during the assembly and eruption of a super-sized magma body. Contrib. Mineral. Petrol..

[52] Di Roberto A (2023). Cryptotephras in the marine sediment record of the Edisto Inlet, Ross Sea: Implications for the volcanology and tephrochronology of northern Victoria Land, Antarctica. Quat. Sci. Adv..

[53] Giordano G, Lucci F, Phillips D, Cozzupoli D, Runci V (2012). Stratigraphy, geochronology and evolution of the Mt. Melbourne volcanic field (North Victoria Land, Antarctica). Bull. Volcanol..

[54] Del Carlo P (2022). Tephrostratigraphy of proximal pyroclastic sequences at Mount Melbourne (northern Victoria Land, Antarctica): Insights into the volcanic activity since the last glacial period. J. Volcanol. Geotherm. Res..

[55] Goldstein JI (2003). Scanning Electron Microscopy and X-Ray Microanalysis.

[56] Nielson CH, Sigurdsson H (1981). Quantitative methods for electron microprobe analysis of sodium in natural and synthetic glasses. Am. Miner..

[57] Lavigne F (2013). Source of the great A.D. 1257 mystery eruption unveiled, Samalas volcano, Rinjani Volcanic Complex, Indonesia. Proc. Natl. Acad. Sci. USA.

[58] Hopkins JL (2021). TephraNZ: A major- and trace-element reference dataset for glass-shard analyses from prominent Quaternary rhyolitic tephras in New Zealand and implications for correlation. Geochronology.

[59] Pearson C, Sbonias K, Tzachili I, Heaton TJ (2023). Olive shrub buried on Therasia supports a mid-16th century BCE date for the Thera eruption. Sci. Rep..

[60] Hogg AG (2003). A wiggle-match date for Polynesian settlement of New Zealand. Antiquity.

[61] Winstrup M (2012). An automated approach for annual layer counting in ice cores. Clim. Past.

[62] Clarkson BR, Patel RN, Clarkson BD (1988). Composition and structure of forest overwhelmed at Pureora, central North Island, New Zealand, during the Taupo eruption (c. AD 130). J. R. Soc. N. Z..

[63] Palmer JG, Ogden J, Patel RN (1988). A 426-year floating tree-ring chronology from *Phyllocladus trichomanoides* buried by the Taupo eruption at Pureora, central North Island, New Zealand. J. R. Soc. N. Z..

[64] Lane C (2011). Cryptotephra from the 74 ka BP Toba super-eruption in the Billa Surgam caves, southern India. Quat. Sci. Rev..

[65] Barker SJ (2019). Modeling ash dispersal from future eruptions of Taupo supervolcano. Geochem. Geophys. Geosyst..

[66] Neff PD, Bertler NAN (2015). Trajectory modelling of modern dust transport to the Southern ocean and Antarctica. J. Geophys. Res. Atmos..

[67] Toohey M, Sigl M (2017). Volcanic stratospheric sulfur injections and aerosol optical depth from 500 BCE to 1900 CE. Earth Syst. Sci. Data.

[68] Sharpe MS (2022). A sulfur and halogen budget for the large magmatic system beneath Taupō volcano. Contrib. Miner. Petrol..

[69] Dunbar NW, Zielinski GA, Voisins DT (2003). Tephra layers in the Siple dome and taylor dome ice cores, Antarctica: Sources and correlations. J. Geophys. Res..

[70] Howat IM, Porter C, Smith BE, Noh M-J, Morin P (2019). The reference elevation model of Antarctica. Cryosphere.

[71] Johnson JS (2022). Review article: Existing and potential evidence for Holocene grounding line retreat and readvance in Antarctica. Cryosphere.

