# Supplementary material for: Volcanic glass from the 1.8 ka Taupō eruption (New Zealand) detected in Antarctic ice at ~ 230 CE
Source: Sci Rep. 2023 Oct 9;13:16720. doi: 10.1038/s41598-023-42602-3 (PMC10562440; doi:10.1038/s41598-023-42602-3)
Supplement: Supplementary file 2 — Supplementary Information 2. [file 41598_2023_42602_MOESM2_ESM.docx]

**Volcanic glass from the 1.8 ka Taupō eruption (New Zealand) detected in Antarctic ice at ~230 CE**

Stephen B. Piva^a,^*, Simon J. Barker^a^, Nels A. Iverson^b^, V. Holly L. Winton^c^, Nancy A. N. Bertler^c,d^, Michael Sigl^e^, Colin J. N. Wilson^a^, Nelia W. Dunbar^b^, Andrei V. Kurbatov^f^, Lionel Carter^c^, Bruce L. A. Charlier^a^, Rewi M. Newnham^a^

^a^ *School of Geography, Environment and Earth Sciences, Victoria University of Wellington, P.O. Box 600, Wellington, 6140, New Zealand*

*^b^ New Mexico Bureau of Geology and Mineral Resources, New Mexico Institute of Mining and Technology, 801 Leroy Place, Socorro, New Mexico, 87801, United States of America*

*^c^ Antarctic Research Centre, Victoria University of Wellington, P. O. Box 600, Wellington, 6140, New Zealand*

*^d^ GNS Science, National Isotope Centre, PO Box 30-368, Lower Hutt, 5040, New Zealand*

*^e^ Climate and Environmental Physics, Physics Institute and Oeschger Centre for Climate Change Research, University of Bern, Hochschulstrasse 4, Bern, 3012, Switzerland*

*^f^ Climate Change Institute, School of Earth and Climate Sciences, University of Maine, 168 College Avenue, Orono, Maine, 04469, United States of America*

*Corresponding author.

*E-mail address:* [stephen.piva@vuw.ac.nz](mailto:stephen.piva@vuw.ac.nz) (S. B. Piva).

**Supplementary File 2**

**Part 1: Supplementary Table and Figures**

**Table S1.** *Characteristics of the WDC06A, SPC14, and RICE ice cores, and their respective drill sites and drilling campaigns. For more information please refer to the references within the cited papers below.*

| **Ice core** | **Years drilled** | **Drill site location** | **Present site characteristics** | **Drill and drilling fluid used** | **Drillers** | **Core dimensions** | **Timescale** |
| --- | --- | --- | --- | --- | --- | --- | --- |
| WDC06A | 2006‑2011 | 79°28’48” S, 112°06’36” W; 1,766 m asl^1^ | ‑30 °C MAT; 22 cm/yr ice accumulation rate | DISC drill; Isopar K/HCFC 141B | IDDO group | 12.2 cm diameter; 3,405 m long | WD2014^[37]^ |
| SPC14 | 2014‑2016 | 89°59’24” S,  98°09’36” W; 2,835 m asl | ‑50 °C  MAT; ~8 cm/yr ice accumulation rate | USA Intermediate Depth Drill; ESTISOL 140 | IDDO group | 9.8 cm diameter; 1,751 m long | SP19^[39]^ |
| RICE | 2011‑2013 | 79°21’36” S, 161°42’36” W; 550 m asl | ‑23.5 °C MAT ~22 cm/yr ice accumulation rate | NZ Intermediate Ice Core Drill; ESTISOL 240‑COASOL | ARC SDO | 763 m long | RICE17^[15,16]^ |

**^1^asl:** above sea level. **^4^IDDO:** Ice Drilling Design and Operations.

**^2^MAT:** Mean Annual Temperature.  **^5^PICO:** Polar Ice Core Office.

**^3^DISC:** Deep Ice Sheet Coring. **^6^ARC SDO:** Antarctic Research Centre’s Science Drilling Office.


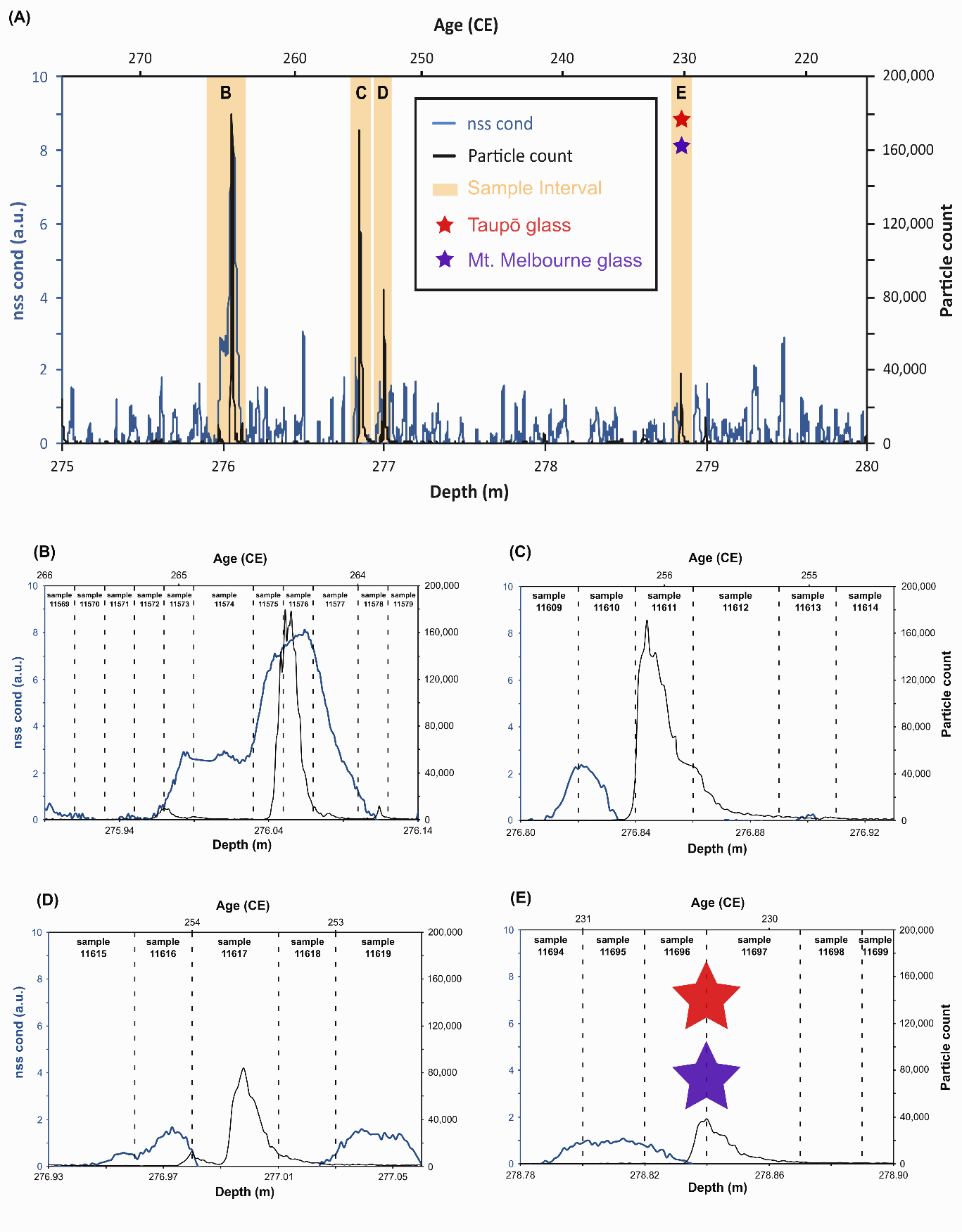


**Figure S1. (A)** Concentrations of nss cond (blue), and particles (black) in the RICE ice core, Antarctica, highlighting in orange the depth intervals of samples **(B‑E)**that were melted, pipetted and geochemically analysed. Microscopic glass derived from the 1.8 ka Taupō eruption (red star) and Mt. Melbourne, Antarctica (purple star) was found in samples 11696 and 11697 of the RICE ice core spanning 278.822 m to 278.866 m depth. Ages are provided from the RICE17 chronology^[15]^.


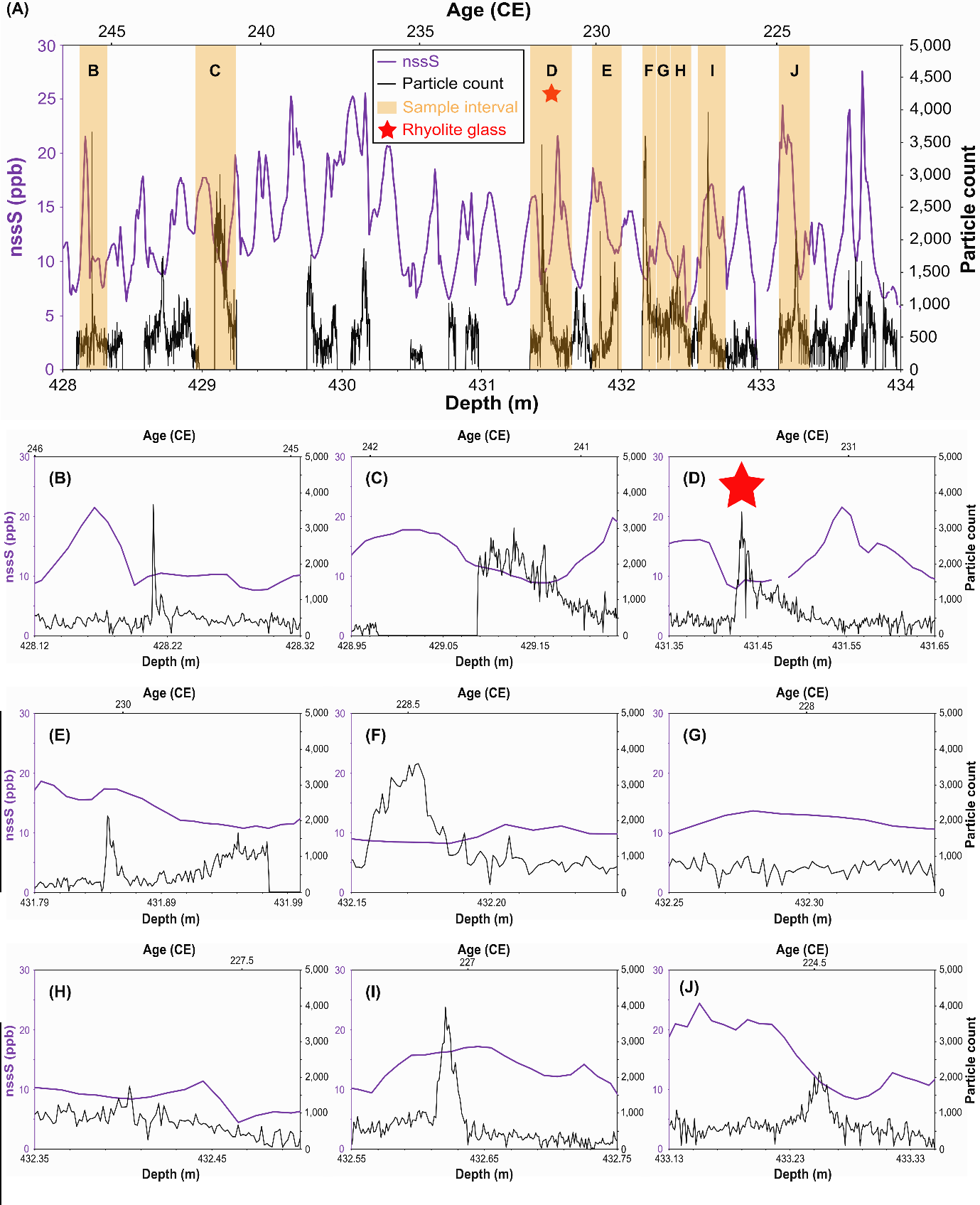


**Figure S2. (A)** Concentrations of nssS (purple), and particles >1.1 µm (black) in the WDC06A ice core, highlighting in orange the depth intervals of samples **(B‑J)**that were melted, filtered and geochemically analysed in search of microscopic glass from the Taupō eruption. A compositionally‑similar rhyolite glass shard (red star) was found in the sample spanning 431.35 m to 431.65 m depth. Ages are provided from the WD2014 chronology^[37]^.
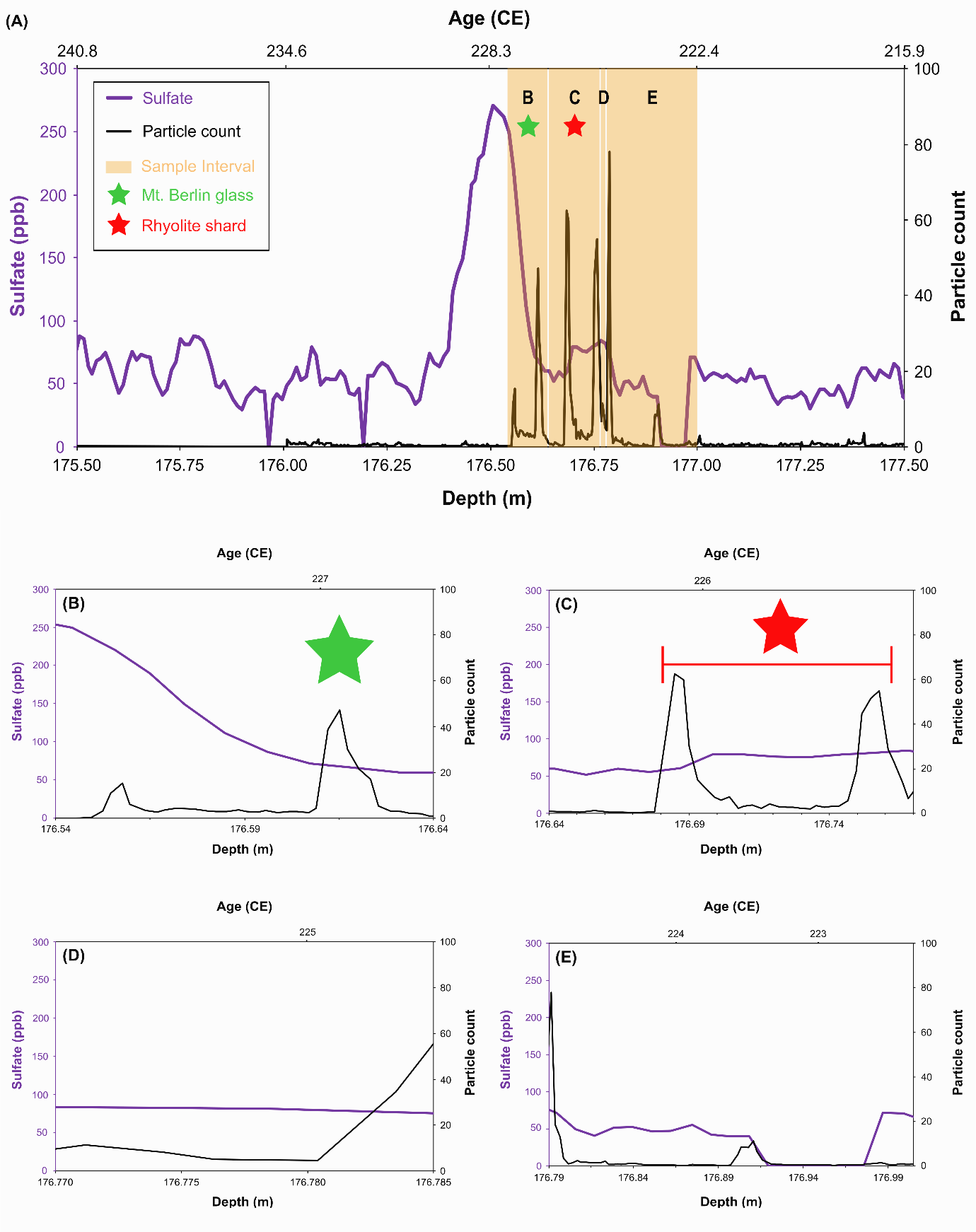


**Figure S3. (A)** Concentrations of sulfate (purple) and particles >10 µm (black) in the SPC14 ice core, highlighting in orange the depth intervals of samples **(B‑E)**that were melted, filtered and geochemically analysed. Microscopic glass derived from Mt. Berlin, Antarctica (green star) was found in the sample spanning 176.54 m to 176.64 m depth, while a rhyolite shard (red star) was identified downcore (176.64‑176.77 m). Ages are provided from the SPC14‑02 chronology.

**
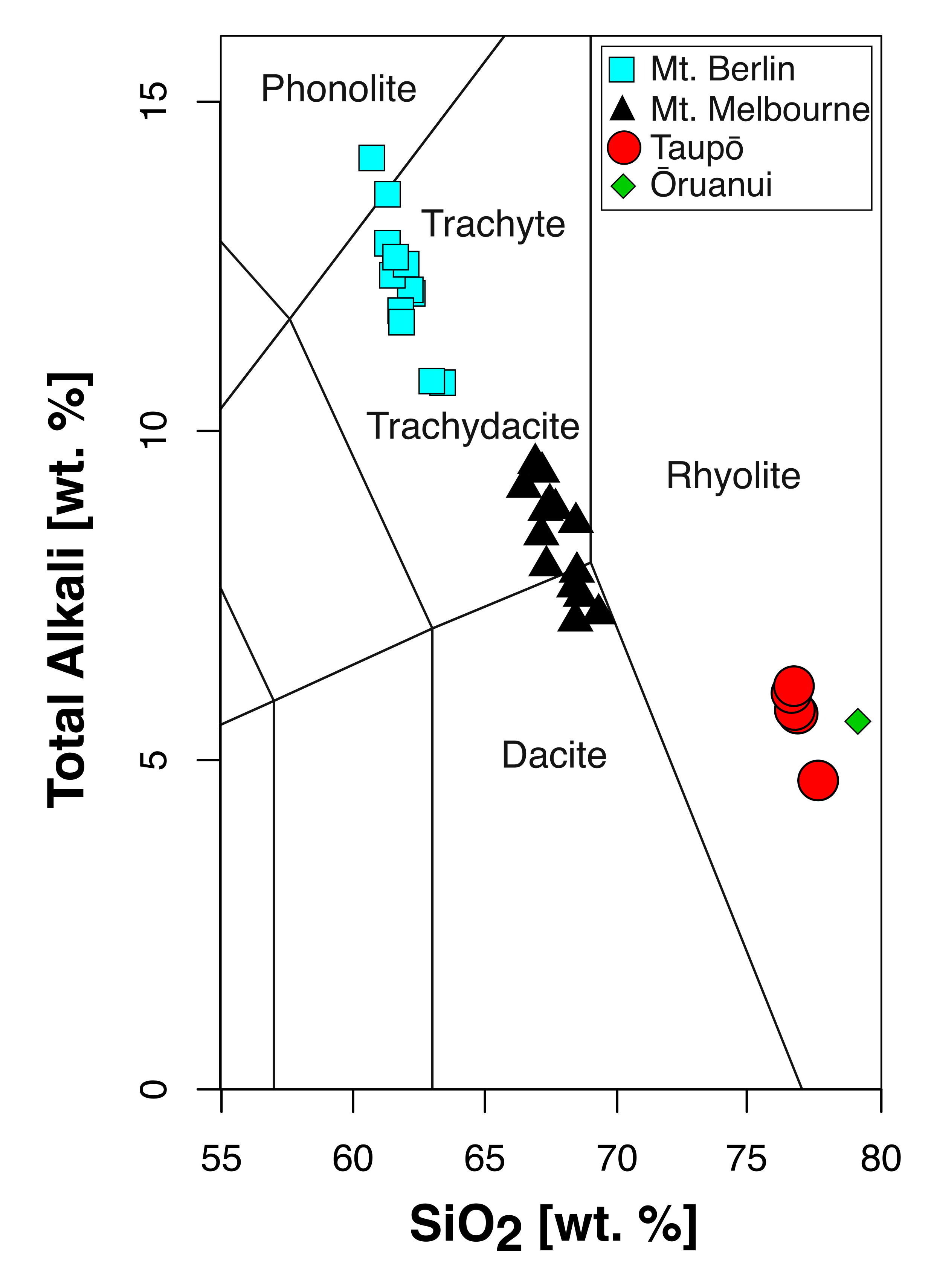
**

**Figure S4.** Total Alkali versus Silica (TAS) classification diagram^[72]^ showing the normalized glass major element compositions, measured by polished EMPA-WDS, of the cryptotephra particles found in the RICE ice core derived from eruptions of Mt. Melbourne (black triangles) and Taupō (red circles; note green diamond is an Ōruanui glass shard) volcanoes, as well as Mt. Berlin‑derived cryptotephra particles (cyan squares) found in the SPC14 ice core.


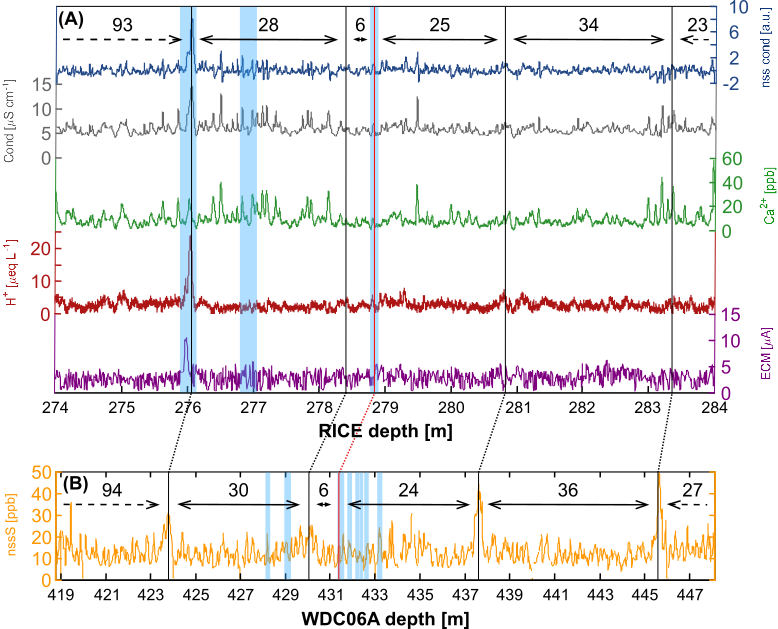


**Figure S5. (A)** The RICE Volcanic proxy records: ECM (purple), acidity (H^+^; red), and nss cond (blue) based on the conductivity‑to‑calcium (conductivity: grey, Ca^2+^: green) excess^[15]^. **(B)** Matching of the RICE records to the WAIS Divide nssS (yellow) record^[7]^, noting the relative position of ice core samples investigated in this study (blue bars). Black vertical lines (solid and dashed) indicate volcanic match points^[15]^, with the number of annual layers between match points in the two records provided according to their respective timescales (RICE17 and WD2014; horizontal arrows). The red vertical lines (solid and dashed) represent the location of the Taupō cryptotephra horizon in RICE and suggested match point in WDC06A.


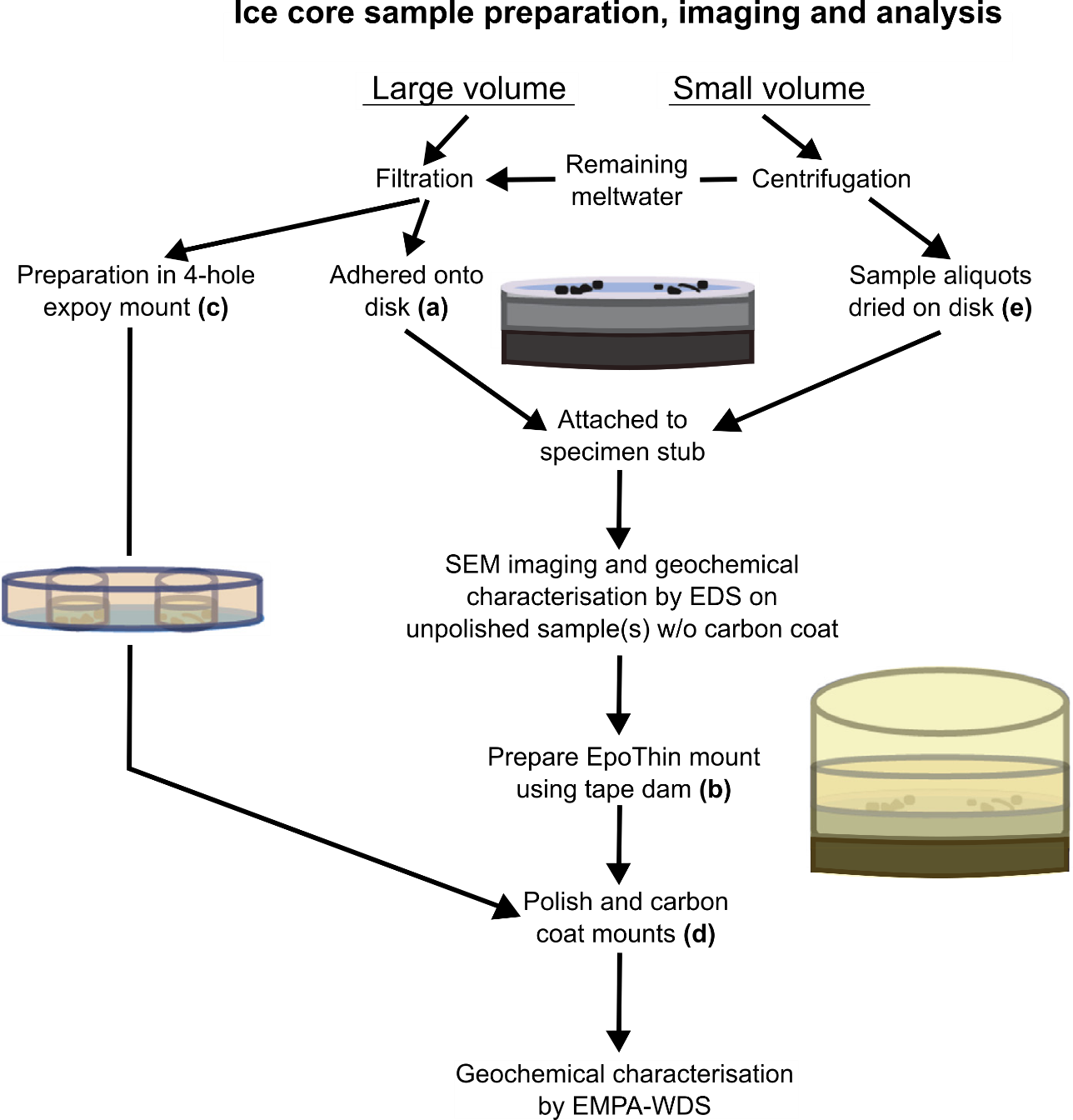


**Figure S6.** Flow chart of how ice core samples were prepared and analysed based on their volume (adapted from Iverson et al. ^[22]^). Small volume (<10 ml) samples were treated differently from large volume samples (>10 ml) to maximise particle extraction. **(A)** After filtration, particulate matter was transferred and adhered onto an ultra‑flat, conductive aluminium disk. For easy handling the disk was attached to a specimen stub prior to SEM imaging and geochemical characterisation by EDS without carbon coating. **(B)** EpoThin mount prepared from disk using a tape dam. **(C)** Sample prepared in parallel, with particulate matter encased in epoxy by backfilling a 4‑hole epoxy mount. **(D)** Mount was polished, and carbon coated prior to EMPA‑WDS. **(E)**After centrifugation, sample aliquots were dried on disk, and remaining meltwater was filtered and archived. If necessary, the filtered particulate matter was prepared in parallel for unpolished SEM‑EDS and polished EMPA‑WDS.

**Part 2: Volcanic Synchronization:**

The ability to resolve and confidently identify annual layers in polar ice cores depends on the measurement resolution and the thickness of annual layers. Ice cores from high snow accumulation sites in Greenland and Antarctica are thus predominantly dated by annual layer counting whereas ice cores from lower snow accumulation sites, or from sites that experience strong layer thinning are often dated using discrete stratigraphic marker horizons. Volcanic synchronization is based on delineating ice core chronologies by identifying common volcanic signatures from volcanic eruptions in ice cores and the subsequent transfer of ages from a reference chronology to the ice core to be dated.

This is a common method in ice core sciences and it has been used to establish lattices of synchronized ice cores extending over the Common Era, Holocene, and Glacial periods^[9,73,74]^. In practice, this is an iterative process, in which starting from clear correlations between extreme volcanic events, smaller and smaller volcanic signal sequences can be identified based on their characteristic relative timing. Independent evaluation for the correctness of the attributed stratigraphic anchor points is based on the evolution of the layer thickness profile, which typically varies only marginally from the long-term mean between neighboring volcanic anchor points. Detailed descriptions on volcanic synchronization techniques are provided by Sigl et al. ^[74]^ and Buizert et al. ^[75]^.

Here, we used the non‑sea‑salt sulfur (nssS) and non‑sea‑salt sulfate (nssSO_4_^2-^) concentration records from WDC06A^[4]^ on the WD2014 chronology^[37]^ as a reference record, and the acidity (H^+^) concentration record from RICE^[15]^ (complemented by liquid conductivity and calculated non‑sea‑salt conductivity [nss cond] records for intervals where the H^+^ data was missing). We applied volcanic synchronization against WDC06A to RICE allowing for a total of 74 volcanic matches with WDC06A going back to 500 BCE (**Figs. S7-S11; Table S2**). The only prior information was the age of the top of the records (1894 CE) and an initial estimate of the mean accumulation rate (0.123 m water equivalent per year). We attribute two prominent large volcanic signatures in RICE at 164.07 m and 325.26 m to the two largest eruptions of the past 2,500 years of Samalas in 1257 CE and an unidentified eruption of similar strength in 426 BCE. The former match-point is further supported by an ice core tephra correlation both in WDC06A and RICE to an eruption of Rittman volcano dated 1252 ± 2 CE (95 % confidence) in the WD2014 chronology^[40]^. Based on these two initial volcanic tie-points we deemed three additional volcanic tie-points (248.77m, 252.09m, and 276.06m) as reliable age markers, dated 574, 540, and 266 CE in the WD2014 age model. All three signals are considered to derive from major stratospheric eruptions with global aerosol dispersal (often called “bipolar events”)^[7,67,76]^ and thus their correlative signatures in Greenland ice cores can provide independent ages (e.g., based on annual layer counting). The volcanic match-point closest to the stratigraphic position of the Taupō cryptotephra in RICE is dated 231 ± 3 CE (95 % confidence) in the WD2014 chronology, which is one year younger than the position of the RICE tephra layer attributed to the Taupō eruption, and likely related to the sulfuric acid deposition from this eruption peaking later than the deposition of the cryptotephra. Conditional to the correctness of the volcanic synchronization at this time period, the date of the Taupō eruption in WD2014 is 230 ± 3 CE.

Based on the available annual layer counted chronologies WD2014^[37]^, GICC21^[77]^, NGRIP‑DRI^[78]^, and NS1-2011^[7]^, and existing temperature reconstructions based on tree‑rings^[79-81]^, we also provide a best estimate of the absolute ages and their uncertainty for the five “bipolar events” mentioned above, and combine these with the sequential annual layer counting uncertainty from the RICE17 chronology^[15]^ relative to the RICE ice core section containing the Taupō cryptotephra (**Table S3**) . We assume these two age uncertainties to be independent and use their root sum square as the total uncertainty estimate for the year of the Taupō eruption. The results vary depending on the starting point for annual layer counting between 210 and 242 CE, with a mean age of 231 CE and a standard error of the mean of ± 6 years. The variations may be due to potentially incorrect volcanic synchronization for the bipolar events or may reflect underestimated errors in the annual layer interpretation. A comparison of the annual layer count between these five bipolar markers indicates undercounting of annual layers in deeper sections consistent with many previous age models for deep ice cores which tended to undercount annual layers when annual layer thicknesses rapidly decreased. Such undercounting can be expected for the RICE chronology, given the rapidly decreasing thicknesses of annual layers with increasing age (i.e., 6 cm ice equivalent per year in 426 BCE *versus* 16 cm ice equivalent per year in 1257 CE).

Finally, as a third age estimate for the Taupō eruption we consider the annual layer counting uncertainties derived by the StratiCounter^[15]^ to realistically represent annual layer interpretation uncertainties. The RICE17 age model gives a 2σ age uncertainty of ± 13 years for the RICE ice core sections containing fallout from the Samalas 1257 CE eruption, which increased to ± 32 years for the RICE ice core section containing Taupō cryptotephra. If we subtracted the ice core age uncertainty of ± 13 years that had accumulated until the known age of the Samalas eruption, the absolute age of the Taupō cryptotephra layer in RICE17 is 230 ± 19 CE (95 % confidence).


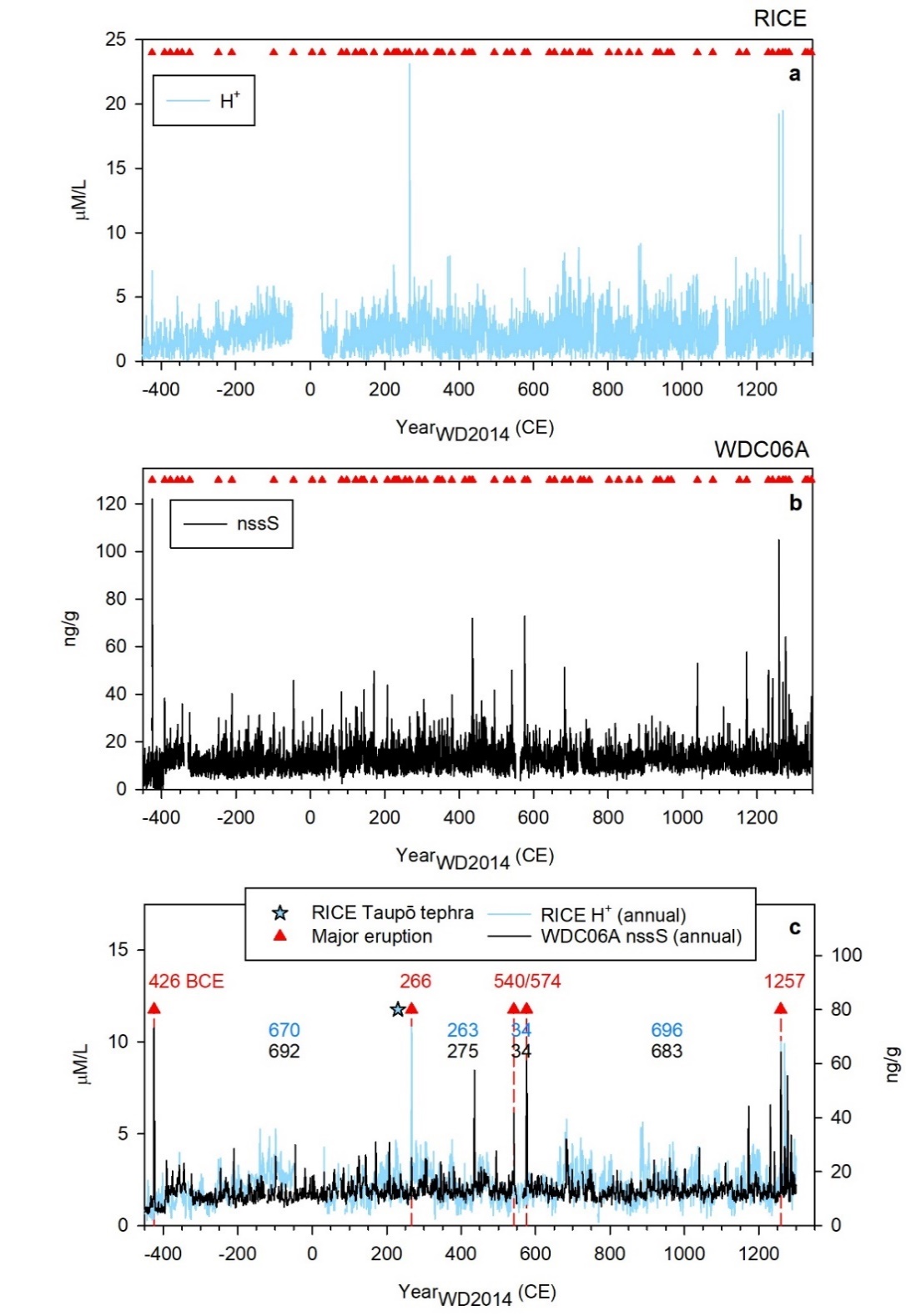


**Figure S7. (A)** RICE acidity [H^+^] record between 450 BCE and 1350 CE^[15]^, red triangles are common volcanic markers with WDC06A. **(B)** WDC06A non-sea-salt sulfur [nssS] record^[4,7]^, triangles are common volcanic markers with RICE. Note that before 394 BCE [nssS] is based on dividing measured non‑sea‑salt sulfate [nssSO_4_] by 3. **(C)** Annual mean RICE [H^+^] and WDC06A [nssS] with five major bipolar eruptions indicated (red triangles) and position of Taupō cryptotephra marked (blue star). The number of annual layers in between the five volcanic markers is provided using the annual layer counted chronologies RICE17^[15]^ (blue) and WD2014^[37]^ (black).


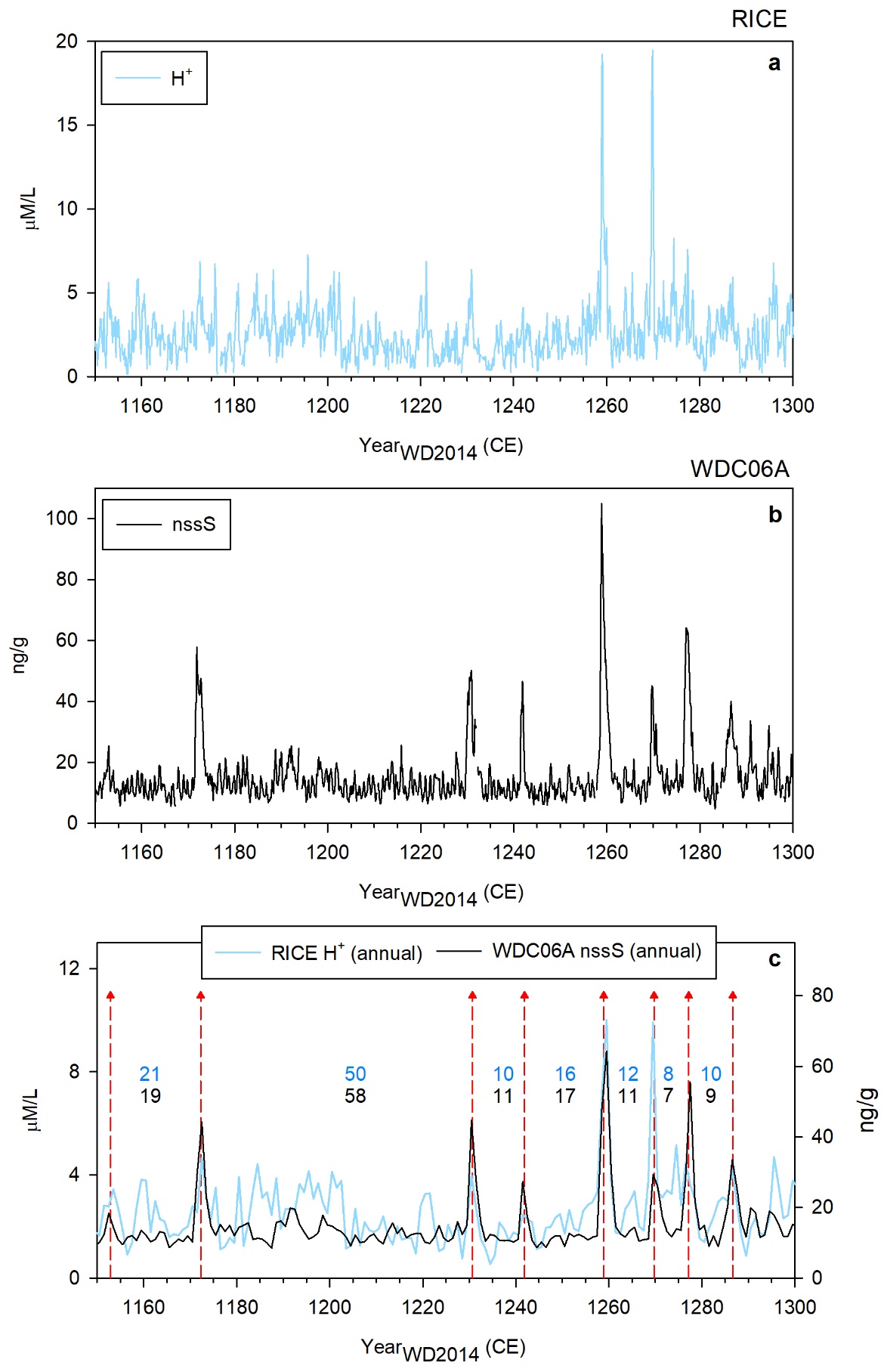


**Figure S8.** As Figure S7 but for 1150–1300 CE.


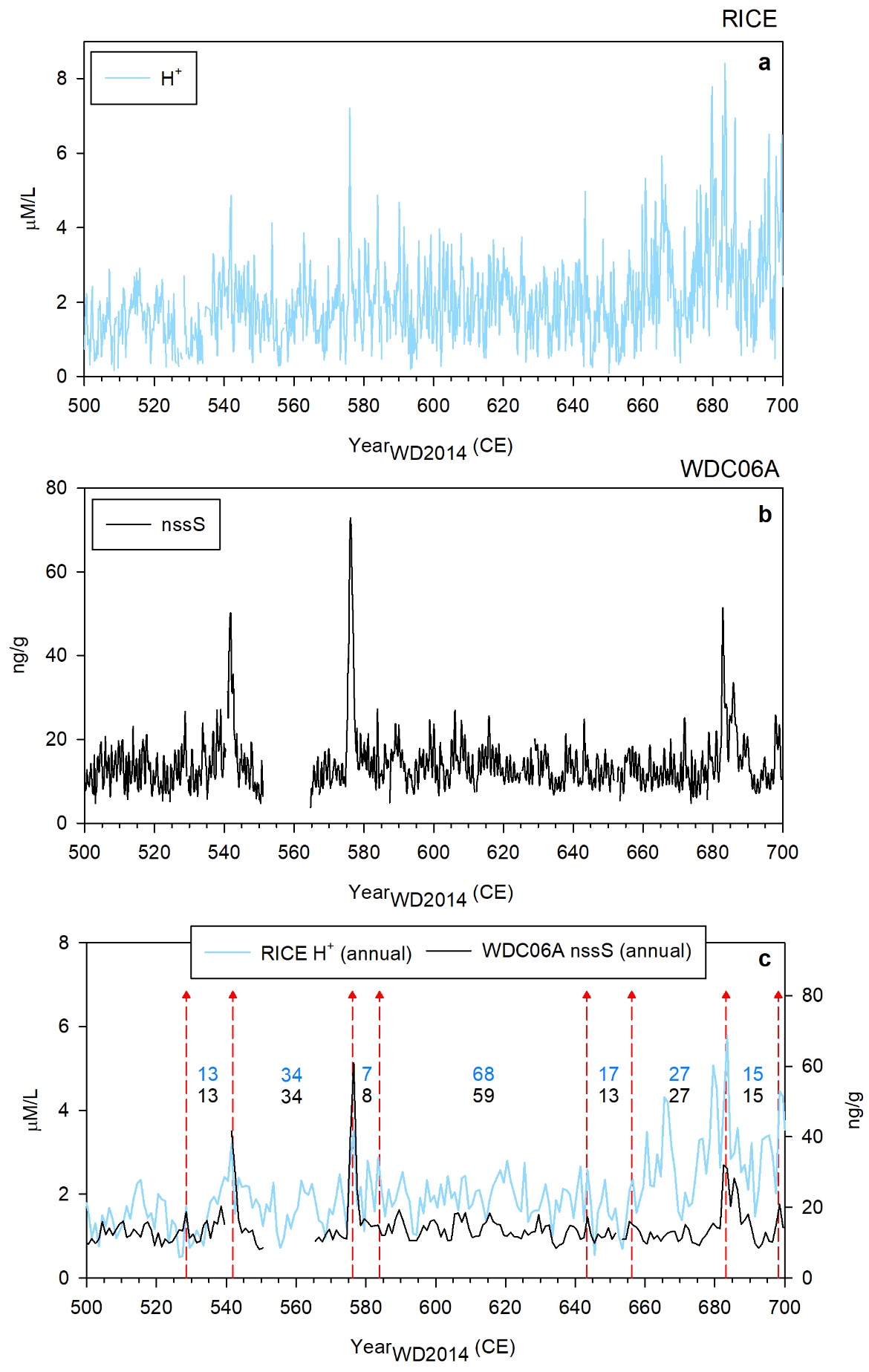


**Figure S9.** As Figure S7 but for 500–700 CE.


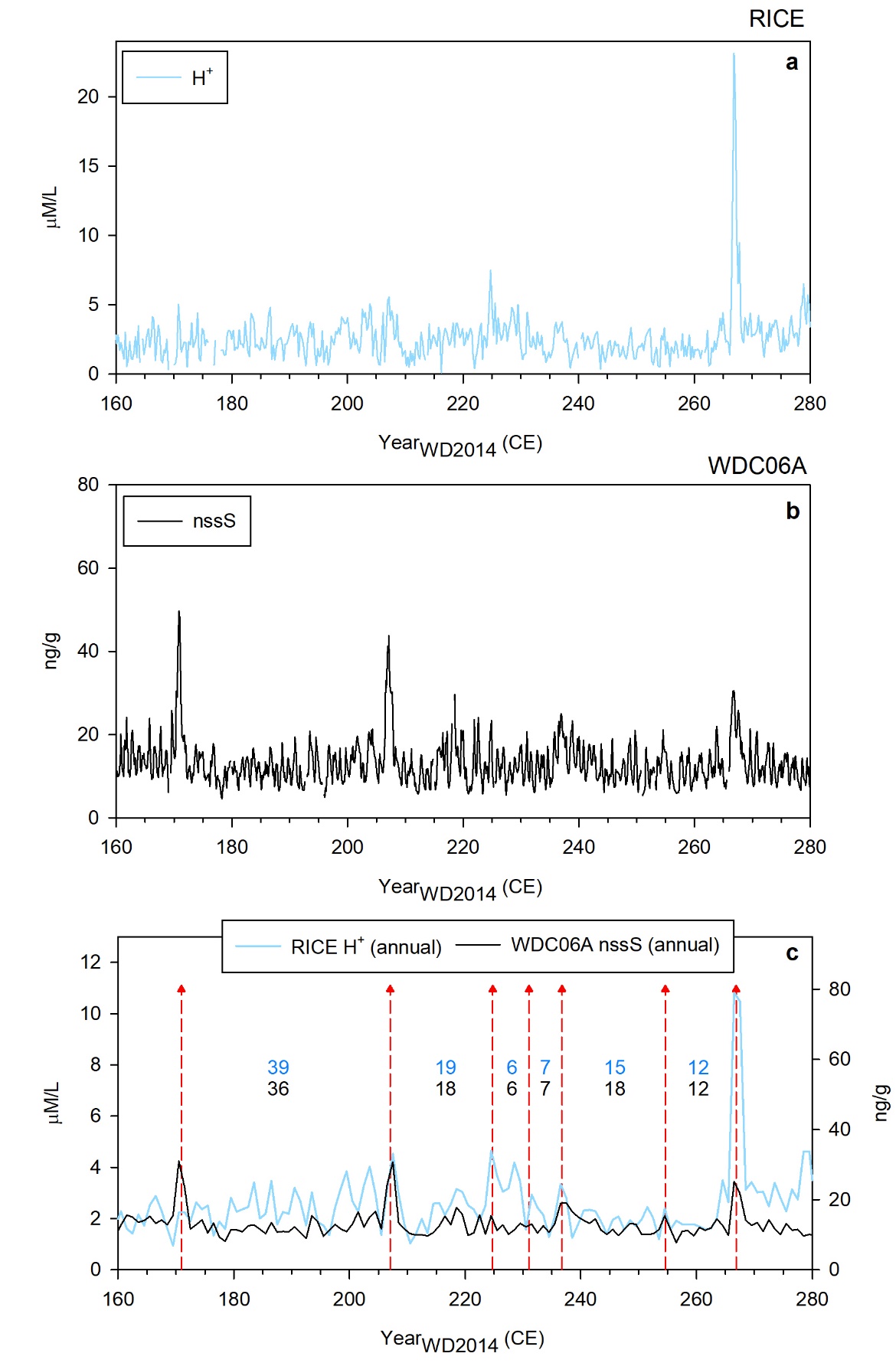


**Figure S10.** As Figure S7 but for 160–280 CE.


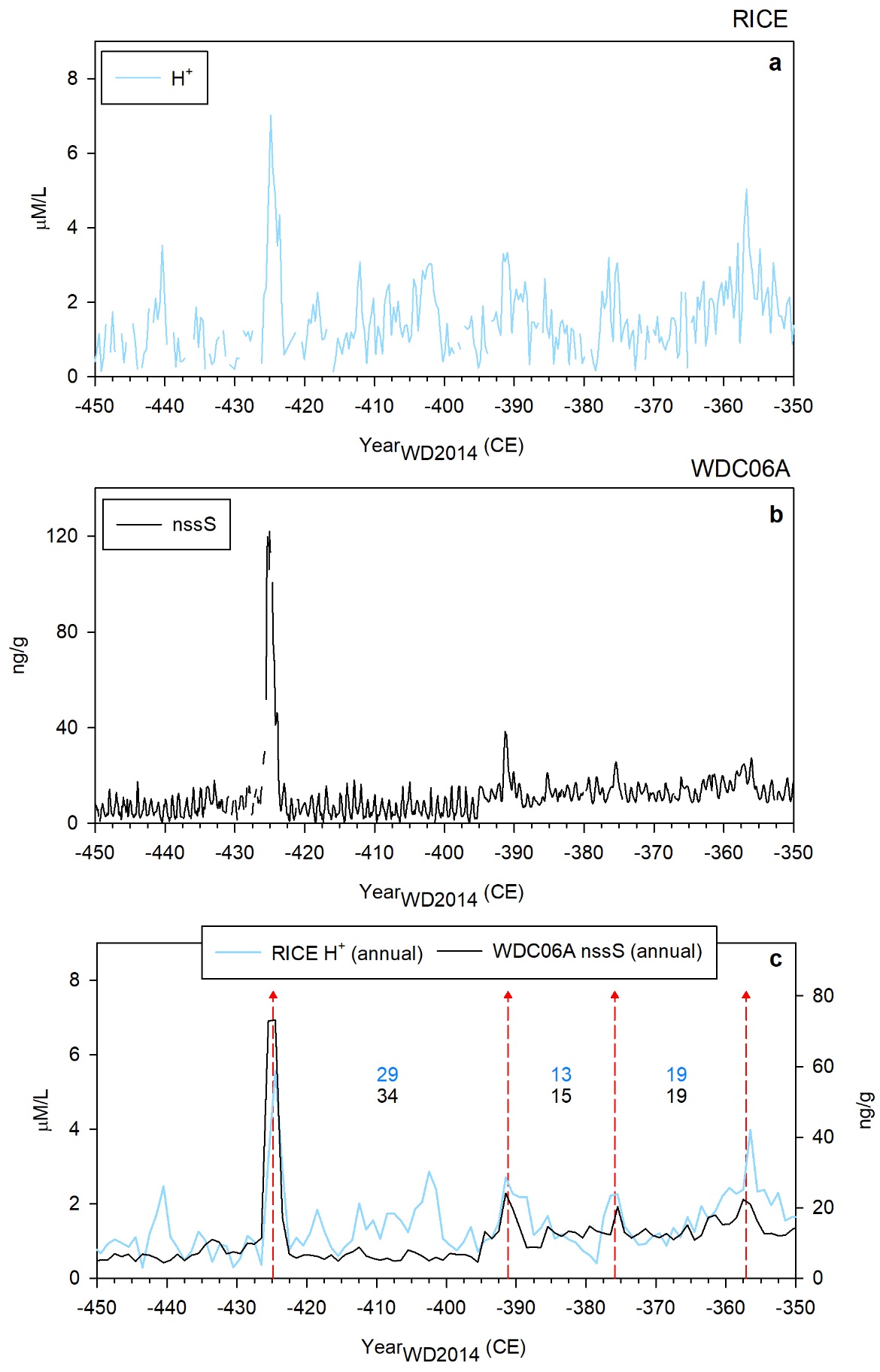


**Figure S11.** As Figure S7 but for 450–350 BCE.

**Table S2.** Volcanic synchronization of RICE and WDC06A

| **Match‑point type (acid, cond)** | **WDC06A**  **(m)** | **Age WD2014 (CE)** | **WD2014 Age (95 % CI))** | **RICE (m)** | **RICE17 Age (CE)** | **RICE17 Age (95 % CI))** | **RICE dz/dt (m/yr)** |
| --- | --- | --- | --- | --- | --- | --- | --- |
| Start | 38.13 | 1894.0 | 1 | 40.00 | 1894.4 | 2 | N/A |
| acid | 46.49 | 1864.1 | 1 | 47.89 | 1863.4 | 3 | 0.264 |
| acid | 72.36 | 1762.7 | 1 | 69.63 | 1770.1 | 6 | 0.214 |
| acid | 88.06 | 1694.9 | 1 | 84.01 | 1704.2 | 7 | 0.212 |
| acid | 92.57 | 1674.8 | 1 | 88.85 | 1680.3 | 7 | 0.241 |
| acid | 110.26 | 1600.9 | 1 | 102.82 | 1613.9 | 8 | 0.189 |
| acid, cond | 142.90 | 1460.0 | 2 | 131.04 | 1458.4 | 11 | 0.200 |
| acid | 161.49 | 1378.7 | 2 | 145.07 | 1376.5 | 11 | 0.173 |
| acid, cond | 168.99 | 1346.0 | 2 | 150.51 | 1345.3 | 12 | 0.166 |
| cond | 171.06 | 1336.6 | 2 | 152.08 | 1334.3 | 12 | 0.165 |
| acid | 172.43 | 1330.7 | 2 | 153.06 | 1328.4 | 12 | 0.165 |
| acid | 182.54 | 1286.6 | 2 | 159.51 | 1287.3 | 13 | 0.147 |
| acid | 184.61 | 1277.1 | 2 | 160.98 | 1277.7 | 13 | 0.155 |
| acid | 186.19 | 1269.7 | 2 | 162.18 | 1269.7 | 13 | 0.162 |
| **acid, cond** | **188.69^a^** | **1258.9^a^** | **1** | **164.07** | **1257.3** | **13** | **0.174** |
| acid | 192.78 | 1241.9 | 2 | 166.82 | 1241.4 | 13 | 0.162 |
| acid | 195.33 | 1230.7 | 2 | 168.35 | 1231.2 | 14 | 0.137 |
| acid | 208.92 | 1172.3 | 2 | 176.03 | 1180.9 | 16 | 0.131 |
| acid | 213.36 | 1152.9 | 2 | 178.75 | 1160.1 | 17 | 0.140 |
| acid | 229.79 | 1081.8 | 2 | 188.94 | 1089.8 | 18 | 0.143 |
| acid | 240.08 | 1040.0 | 2 | 194.81 | 1043.2 | 19 | 0.141 |
| acid | 256.92 | 969.6 | 2 | 203.43 | 974.6 | 20 | 0.122 |
| acid | 259.07 | 961.0 | 2 | 204.44 | 966.2 | 20 | 0.117 |
| acid | 264.18 | 939.7 | 2 | 207.09 | 945.3 | 20 | 0.125 |
| acid | 266.94 | 929.6 | 2 | 208.13 | 937.0 | 20 | 0.102 |
| acid | 278.15 | 883.1 | 2 | 213.51 | 891.2 | 21 | 0.116 |
| acid | 284.33 | 857.6 | 2 | 216.43 | 867.7 | 21 | 0.115 |
| acid | 291.00 | 828.8 | 2 | 220.01 | 838.5 | 21 | 0.124 |
| cond | 296.91 | 802.8 | 2 | 223.11 | 812.0 | 21 | 0.119 |
| acid | 309.64 | 749.6 | 2 | 229.36 | 751.9 | 22 | 0.118 |
| acid | 313.15 | 734.8 | 2 | 231.10 | 735.0 | 22 | 0.118 |
| acid | 315.38 | 725.9 | 2 | 232.09 | 726.1 | 22 | 0.111 |
| acid | 322.31 | 698.2 | 2 | 235.54 | 695.0 | 23 | 0.124 |
| acid, cond | 325.81 | 683.2 | 2 | 237.30 | 679.5 | 23 | 0.118 |
| acid | 332.27 | 656.2 | 2 | 240.37 | 652.9 | 24 | 0.114 |
| acid, cond | 335.47 | 643.3 | 2 | 241.98 | 636.1 | 26 | 0.124 |
| acid | 349.72 | 583.9 | 2 | 248.13 | 568.1 | 28 | 0.104 |
| **acid** | **351.42^b^** | **576.1^b^** | **2** | **248.77** | **561.2** | **28** | **0.082** |
| **acid** | **359.70^b^** | **541.9^b^** | **3** | **252.09** | **527.3** | **28** | **0.097** |
| acid | 362.95 | 528.6 | 3 | 253.23 | 514.1 | 29 | 0.086 |
| acid | 370.99 | 495.0 | 3 | 256.25 | 481.3 | 29 | 0.090 |
| acid | 384.68 | 435.4 | 3 | 261.87 | 419.5 | 30 | 0.094 |
| acid | 386.92 | 426.8 | 3 | 262.70 | 411.2 | 30 | 0.096 |
| acid | 389.71 | 415.3 | 3 | 263.67 | 399.8 | 30 | 0.084 |
| acid | 398.01 | 379.7 | 3 | 266.63 | 365.0 | 31 | 0.083 |
| acid, cond | 404.16 | 353.9 | 3 | 268.70 | 341.3 | 31 | 0.080 |
| acid | 406.10 | 345.5 | 3 | 269.51 | 332.5 | 31 | 0.096 |
| acid | 407.38 | 340.8 | 3 | 269.91 | 328.4 | 31 | 0.086 |
| acid | 414.92 | 307.5 | 3 | 272.53 | 303.0 | 31 | 0.078 |
| acid | 418.49 | 291.7 | 3 | 273.96 | 285.1 | 32 | 0.091 |
| **acid, cond** | **423.73^b^** | **266.8^b^** | **3** | **276.06** | **264.3** | **32** | **0.085** |
| acid | 426.19 | 254.6 | 3 | 277.08 | 252.4 | 32 | 0.083 |
| acid | 430.12 | 236.8 | 3 | 278.37 | 236.9 | 32 | 0.072 |
| acid | 431.54 | 231.0 | 3 | 278.82 | 230.6 | 32 | 0.080 |
| **RICE Taupō cryptotephra** | **431.80^c^** | **230.1^c^** | **3** | **278.90** | **229.7** | **32** | **0.076** |
| acid | 433.18 | 224.8 | 3 | 279.30 | 224.3 | 32 | 0.076 |
| acid | 437.61 | 207.0 | 3 | 280.83 | 205.1 | 33 | 0.086 |
| acid | 445.61 | 170.9 | 3 | 283.65 | 166.1 | 35 | 0.078 |
| acid | 451.87 | 143.9 | 3 | 285.83 | 136.2 | 35 | 0.081 |
| acid | 453.26 | 137.0 | 3 | 286.41 | 128.0 | 35 | 0.084 |
| acid | 456.57 | 121.9 | 4 | 287.49 | 113.4 | 36 | 0.071 |
| acid | 462.26 | 97.8 | 4 | 289.16 | 91.1 | 36 | 0.069 |
| cond | 465.67 | 84.0 | 4 | 290.12 | 77.9 | 37 | 0.070 |
| cond | 477.74 | 31.7 | 4 | 293.93 | 27.2 | 37 | 0.073 |
| cond | 483.78 | 5.0 | 4 | 296.01 | 4.0 | 37 | 0.078 |
| cond | 495.11 | -45.3 | 4 | 299.75 | -40.4 | 38 | 0.074 |
| acid, cond | 507.61 | -98.3 | 4 | 303.80 | -94.3 | 39 | 0.076 |
| acid | 532.90 | -210.9 | 4 | 312.14 | -220.4 | 40 | 0.074 |
| acid | 541.63 | -247.1 | 4 | 314.03 | -248.0 | 41 | 0.052 |
| acid | 560.48 | -324.3 | 5 | 318.92 | -319.0 | 42 | 0.063 |
| acid | 565.32 | -344.4 | 5 | 320.22 | -333.9 | 42 | 0.065 |
| acid | 568.06 | -357.1 | 5 | 320.88 | -344.7 | 42 | 0.052 |
| acid | 572.35 | -375.9 | 5 | 322.18 | -363.3 | 42 | 0.069 |
| acid | 576.08 | -391.2 | 5 | 323.15 | -376.8 | 42 | 0.063 |
| **acid** | **583.87^a^** | **-424.8^a^** | **5** | **325.26** | **-405.8** | **42** | **0.063** |
| End | 590.46 | -453.0 | 5 | 327.00 | -430.1 | 42 | 0.062 |

^a^Largest volcanic eruptions of the past 2,500 years dated to 1257 CE (Samalas, Indonesia) and 426 ± 1 BCE (unidentified source); ^b^Four major bipolar volcanic signals from unidentified sources dating 574, 540 and 266 CE a; ^c^RICE layer containing Taupō cryptotephra, estimated corresponding depth and age in WDC06A using linear interpolation between the two neighbouring volcanic match‑points.

**Table S3.** Age and uncertainty estimates (95 % confidence) for volcanic eruptions including Taupō.

| Chronology | Samalas  Year | UE 574  Year | UE 540  Year | UE 267  Year | UE 426 BCE  Year |
| --- | --- | --- | --- | --- | --- |
| WD2014 | 1258 (±1) | 575 (±2) | 541 (±3) | 266 (±3) | 426 BCE (±5) |
| NS1-2011 | 1258 (±1) | 574 (±2) | 540 (±1) | 263 (±3) | 426 BCE (±5) |
| GICC21 | 1258 (±2) | 574 (±5) | 540 (±5) | 267 (±5) | 425 BCE (±6) |
| NGRIP2-DRI | 1259 (±1) | 574 (±2) | 540 (±1) | 264 (±2) | 426 BCE (±3) |
| Ice core mean | 1258 CE | 574 CE | 540 CE | 265 CE | 426 BCE |
| 2σ | 1.0 | 1.0 | 1.0 | 3.7 | 1.0 |
| Büntgen20 | 1258 | 575 | 541 | 268 & 271 | N/A |
| Luterbacher16 | 1258 | 574 | 541 | 267-271 | N/A |
| Salzer07 | 1257/1259 | 574 | 541 | 268 & 273 | 426 BCE |
| Best eruption age | 1257 (±0) | 574 (±1) | 540 (±1) | 266 (±2) | 426 BCE (±1) |
| RICE17 annual layers  wrt Taupō layer | 1018 (±20) | 332 (±6) | 298 (±4) | 35 (±2) | 635 (±10) |
| RICE17 age CE | 230 (±20) | 242 (±6) | 242 (±6) | 231 (±2) | 210 (±10) |
| Taupō mean age | 231 CE | | | | |
| Taupō age uncertainty (2σ) | 26 years | | | | |

**Additional References**

1. LeBas, M. J., Le Maitre, R. W., Streckeisen, A., Zanettin, B. & IUGS Subcommission on the Systematics of Igneous Rocks. A chemical classification of volcanic rocks based on the total alkali-silica diagram. *J. Petrol.* **27**, 745-750 (1986).
2. Seierstad, I. K. *et al.* Consistently dated records from the Greenland GRIP, GISP2 and NGRIP ice cores for the past 104 ka reveal regional millennial-scale δ^18^O gradients with possible Heinrich event imprint. *Quat. Sci. Rev.* **106**, 29-46 (2014).
3. Sigl, M. *et al.* Insights from Antarctica on volcanic forcing during the Common Era. *Nat. Clim. Change* **4**, 693-697 (2014).
4. Buizert, C. *et al.* Abrupt ice-age shifts in southern westerly winds and Antarctic climate forced from the north. *Nature* **563**, 681-685 (2018).
5. Gautier, E. J. *et al.* 2600-years of stratospheric volcanism through sulfate isotopes. *Nat. Commun.* **10**, 466 (2019).
6. Sinnl, G. M. *et al.* A multi-ice-core, annual-layer-counted Greenland ice-core chronology for the last 3800 years: GICC21. *Clim. Past* **18**, 1125-1150 (2022).
7. McConnell, J. R. *et al.* Lead pollution recorded in Greenland ice indicates European emissions tracked plagues, wars, and imperial expansion during antiquity. *Proc. Natl. Acad. Sci. USA* **115**, 5726-5731 (2018).
8. Büntgen, U. *et al.* Prominent role of volcanism in Common Era climate variability and human history. *Dendrochronologia*, **64**, 125757 (2020).
9. Luterbacher, J. *et al.* European summer temperatures since Roman times*. Environ. Res. Lett.* **11**, 024001 (2016).
10. Salzer, M. W. & Hughes, M. K. Bristlecone pine tree rings and volcanic eruptions over the last 5000 yr. *Quat. Res.* **67**, 57-68 (2007).
